# Decoding the lncRNAome Across Diverse Cellular Stresses Reveals Core p53-effector Pan-cancer Suppressive lncRNAs

**DOI:** 10.1158/2767-9764.CRC-22-0473

**Published:** 2023-05-11

**Authors:** Ramkrishna Mitra, Clare M. Adams, Christine M. Eischen

**Affiliations:** 1 Department of Pharmacology, Physiology, and Cancer Biology, Sidney Kimmel Cancer Center, Thomas Jefferson University, Philadelphia, Pennsylvania.

## Abstract

Despite long non-coding RNAs (lncRNAs) emerging as key contributors to malignancies, their transcriptional regulation, tissue-type expression under different conditions, and functions remain largely unknown. Developing a combined computational and experimental framework, which integrates pan-cancer RNAi/CRISPR screens, and genomic, epigenetic, and expression profiles (including single-cell RNA sequencing), we report across multiple cancers, core p53-transcriptionally regulated lncRNAs, which were thought to be primarily cell/tissue-specific. These lncRNAs were consistently directly transactivated by p53 with different cellular stresses in multiple cell types and associated with pan-cancer cell survival/growth suppression and patient survival. Our prediction results were verified through independent validation datasets, our own patient cohort, and cancer cell experiments. Moreover, a top predicted p53-effector tumor-suppressive lncRNA (we termed *PTSL*) inhibited cell proliferation and colony formation by modulating the G_2_ regulatory network, causing G_2_ cell-cycle arrest. Therefore, our results elucidated previously unreported, high-confidence core p53-targeted lncRNAs that suppress tumorigenesis across cell types and stresses.

**Significance:**

Identification of pan-cancer suppressive lncRNAs transcriptionally regulated by p53 across different cellular stresses by integrating multilayered high-throughput molecular profiles. This study provides critical new insights into the p53 tumor suppressor by revealing the lncRNAs in the p53 cell-cycle regulatory network and their impact on cancer cell growth and patient survival.

## Introduction

The p53 tumor-suppressive transcription factor (TF) is activated in response to various cellular stresses (1). Activated p53 binds to promoter sequences, transactivating thousands of genes, but only a fraction were shown critical for cellular processes that impact tumorigenesis (1, 2). Chromatin immunoprecipitation with sequencing (ChIP-seq) technology has revealed genome-wide p53-binding sites following diverse cellular stresses (2–5). Combining ChIP-seq with gene expression data and evaluating multiple independent studies through meta-analysis led to the identification of high-confidence gene targets of p53 and other TFs (2, 6, 7). Although many protein-coding genes from these approaches were identified as p53 targets, they cannot fully explain the tumor-suppressive effects of p53 activation, suggesting that non–protein-coding genes are likely to have a significant role.

Similar to protein-coding genes, p53 regulates the expression of different types of non-coding RNA, including long non-coding RNA (lncRNA; ref. 8). Although the functions of most of the approximately 16,000 annotated human lncRNAs (9) are unknown, reports indicate lncRNAs have critical roles in cancer and other diseases (10). These lncRNAs impact a diverse range of cellular processes, including those regulated by p53 or triggered after p53 activation. Although lncRNA research is increasing understanding of p53 expression and function, lncRNAs are thought to be cell type-specific (11) and investigated as p53 targets in only one or two cancer types. For example, direct p53 transcriptional lncRNA targets (e.g., *NBAT1*, *PURPL*, *PR-lncRNA-1*, *PR-lncRNA-10*, *PINCR*, *OIP5-AS1*, *LINC00475*) regulate p53 expression levels and activity in specific cancer types (12–19). Other lncRNAs, *GUARDIN*/*LNCTAM34A* and *TP53TG1*, that are direct p53 targets are important for genome stability (20) and the p53 response to DNA damage (21), respectively, in colorectal cancer. In addition, *PICART1*, *ST7-AS1*, *NEAT1*, *Loc285194/TUSC7*, and other lncRNAs are direct p53 targets and have roles in suppressing cancer cell proliferation, migration, invasion, tumor formation and/or other protumorigenic p53 functions in one or two malignancies (22–26). Therefore, because p53 functions as a tumor suppressor across cell types, we investigated whether a core set of p53-effector lncRNAs exist, similar to the core p53-effector protein-coding genes (1, 6), that are recurrently induced by activated p53 and that consequently, suppress tumorigenesis across cell types.

Here, we performed a large-scale data analysis that leveraged multiple types of omics data for 10 different cancer types. Combining our integrative bioinformatics approach with experimental validation revealed previously unidentified p53-effector lncRNAs that likely suppress known cancer hallmark processes across multiple cancers. RNA sequencing (RNA-seq) coupled with network analysis revealed significant associations between the top predicted p53-effector lncRNAs and cell cycle–linked genes across multiple cancers, which was corroborated with qRT-PCR and protein analyses. We verified the induction and tumor-suppressive functions of a top predicted lncRNA in lung adenocarcinoma (LUAD) cells upon p53 activation and its downregulation in our LUAD patient cohort that correlates with poor overall survival across cancer types. Our study reveals a core set of p53-regulated lncRNAs in the non–protein-coding genome that contribute to the pan-tumor suppressive functions of p53 through cell-cycle regulation.

## Materials and Methods

### RNA-seq Data Analysis

From Gene Expression Omnibus (GEO; ref. 27) database, we collected 14 RNA-seq datasets covering 10 different cell lines or eight different cell types (Supplementary Table S1). Samples in seven datasets were treated with the p53-activating agent Nutlin or DMSO vehicle/untreated control. The remaining seven datasets were treated with 5-fluorouracil, doxorubicin, ionizing radiation, or DMSO vehicle/untreated control. We employed FASTQC (version 0.11.8; https://www.bioinformatics.babraham.ac.uk/projects/fastqc/), infer_experiment.py script from RseQC package (28), and the tool Cutadapt (version 2.4; ref. 29) to check the quality and strand specificity, and to trim adapter sequences of the sequencing reads, respectively. Reads were aligned to the human genome (Ensembl version GRCh38) using Hisat2 (30), transcripts were assembled with StringTie (version 1.3.6; ref. 31), and the python script prepDE, present in the StringTie package, was utilized to extract read count information. We used edgeR (32) to normalize the data and compute differential expression of lncRNA and protein-coding genes in the p53-activated cells compared with control cells.

### Identification of Consistently Dysregulated lncRNAs Upon p53 Activation

For individual RNA-seq datasets, we separated the protein-coding genes and lncRNAs into upregulated and downregulated groups, then ranked them based on their expression fold-change values. Rankings of 14 sets of upregulated and 14 sets of downregulated gene/lncRNA lists, obtained from 14 RNA-seq datasets, were subjected to the rank-based meta-analysis method RobustRankAggreg V1.1 (33) to identify consistently dysregulated genes and lncRNAs across the RNA-seq datasets. Under the null hypothesis that the ranked gene/lncRNA lists are uncorrelated, the method assigns *P* values to individual genes/lncRNAs to indicate whether they are consistently upregulated or downregulated across the datasets. To check the stability of the acquired *P* values and minimize false positives, we repeated the same analysis 14 times after excluding one of the rankings, averaged the *P* values, and further corrected the *P* values and computed FDR by the Benjamini–Hochberg (BH) method (34, 35). High-confidence p53-transactivated lncRNAs were determined with the criteria that the lncRNAs had consistent upregulation or downregulation in at least two-thirds of the 14 RNA-seq datasets where they were detected with FDR ≤ 0.05.

### Integrating The Cancer Genome Atlas Genomic, Epigenetic, and Transcriptomic Data

From UCSC Xena database (36), we downloaded mRNA expression, gene copy number, and DNA methylation data for 10 The Cancer Genome Atlas (TCGA) cancer types (Supplementary Table S2). In brief, whole-genome microarray data were used to measure copy-number variation using the GISTIC2 method (37). For DNA methylation, except for ovarian cancer, data from Illumina Infinium HumanMethylation450 platform were used. We used Illumina Infinium HumanMethylation27 for ovarian cancer as a larger set of samples were profiled from this platform. Relative DNA methylation levels were represented as β-values. If a gene's promoter region was linked with multiple methylation probes, we selected a representative probe whose β-values had the strongest negative Spearman rank correlation with the gene's mRNA expression levels (18, 35, 38). We selected one mRNA for downstream analysis if ≥75% of the samples of a cancer type showed normalized expression values of that mRNA ≥ 1. For the same 10 cancer types, we obtained processed lncRNA expression data from the Tanric database (39). We selected one lncRNA for downstream analysis if the 50th and 90th percentile of its normalized expression values were >0 and >0.1, respectively, as described previously (18, 35, 40).

We used normalized expression profiles to measure lncRNA-mRNA expression associations, using either multivariate regression or Spearman rank correlation method. We used *t* tests for measuring significant differential expression of lncRNAs between patient samples with p53 truncating mutations and wild-type p53. The results were further adjusted using the BH multiple test correction method (34).

### Predicting lncRNA-mRNA Associations Using Multivariate Regression

Employing the following multivariate regression analysis, as described before (35), on *n* tumor samples of a given cancer type, we evaluated whether lncRNA (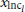) and mRNA (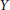) expression association was independent of copy-number variation (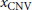) and DNA methylation (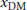) events.



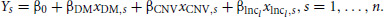



Here β_0_ is the intercept, and β_DM_, β_CNV_, and β_lnc_*l*__ are the regression coefficients that quantify the association strength between modulation of mRNA expression with the modulation of DNA methylation, copy number, and lncRNA expression, respectively. We used FDR *<* 0.001 as the threshold for significantly associated lncRNA-mRNA pairs in each cancer type.

### Identification of Cell Survival/Growth-regulating p53-effector lncRNAs

Genome-scale RNAi screening data on 311 cancer cell lines (Release-DEMETER2 Data v6) and CRISPR screening data on 255 cancer cell lines (Release-Depmap Public 19Q3), together 382 unique cell lines, representing the same cell types as the 10 different cancer tissues extracted from Depmap database (41) were evaluated (Supplementary Table S3). To determine dependency scores for approximately 17,000 genes in a given cell line, algorithms DEMETER2 (42) and CERES (43) were developed for RNAi and CRISPR screens, respectively. For individual cell lines, genes were ranked on the basis of their viability/growth scores determined by these algorithms (41–43) where a lower score corresponds to a lower rank. A gene with a lower rank means a given cell line is more likely to be dependent on that gene for its growth/survival.

We selected 10 major cancer types covering diverse tissue types with lncRNA and gene expression, copy number, and methylation profiles available in TCGA and that also had genome-scale RNAi and CRISPR screening data available in Depmap in >5 cell lines for each of the 10 cancer types. For a specific cancer type *i*, our approach integrated (i) significantly (FDR < 0.001) associated p53-effector lncRNA and mRNA pairs determined from the multivariate regression model and (ii) ranked gene list from RNAi or CRISPR screening on cell lines originating from the primary cancer site *i*, using the gene set enrichment analysis (GSEA; ref. 44) method in the R package WebGestaltR (45). Unlike traditional GSEA where curated pathways are used as gene sets, here we used a group of mRNAs that have significant associations with individual p53-effector lncRNA as the gene set. For a given cell line, our approach implemented the GSEA method on the DEMETER2- or CERES-derived ranked gene list and the gene sets derived from the above multivariate regression model. The gene sets with significantly (FDR < 0.001) negative normalized enrichment scores (NES) indicate their overrepresentation at the bottom of the ranked gene list. A p53-effector lncRNA associated with the gene set with significant negative NES was predicted as a cancer cell survival/growth-regulating lncRNA. For each p53-effector lncRNA, we determined two gene sets that have positive and negative associations with the lncRNA. For a specific cell line, if both positively and negatively associated gene sets of one lncRNA had a significant negative NES, the corresponding lncRNA was not considered for downstream analysis.

### Identifying Genes/lncRNAs Linked to Overall Patient Survival

For a specific gene/lncRNA in a TCGA cancer type, we ranked the samples based on gene/lncRNA expression values. Using the median expression cut-off value, we divided the samples into low or high groups. To evaluate which genes/lncRNAs have a significant impact (|Z-score| > 1.96 that corresponds to *P* < 0.05; log-rank test) on overall patient prognosis in a specific cancer type, we performed univariate survival analysis using log-rank statistical tests (46).

### Cell Culture, Plasmids, and Lentiviral Infection

A549 (CCL-185; RRID:CVCL_0023), H460 (HTB-177; RRID:CVCL_0459), and H1299 (CRL-5803; RRID:CVCL_0060) LUAD cell lines were obtained from and cultured according to ATCC instructions. PC9 (RRID:CVCL_B260) LUAD cells were obtained from Dr. William Pao. Cells were confirmed to be *Mycoplasma* negative (Agilent MycoSensor PCR Assay Kit #302107) and were authenticated by short tandem repeat profiling (ATCC Human Cell STR Profiling). Lentiviral vectors for *PTSL* (*AC087752.3*) and *PSLR-2* were obtained from Vector Builder and *MALAT1* from Addgene (RRID:Addgene_118580). After sequence verification, lentivirus was produced following standard calcium phosphate transfection of 293T cells. One million cells were placed in 10 cm dishes and allowed to adhere overnight (16 hours), prior to being infected. Following a 16-hour incubation with lentivirus, cells were harvested and replated for experimentation.

### RNA Isolation, qRT-PCR, and RNA-seq

For p53 activation experiments, A549 and H460 cells (1 × 10^5^ cells/well) in 6-well plates were treated with Nutlin, cisplatin, or etoposide (compounds from Sigma and used at 10 μmol/L) or were irradiated (5 Gy, cesium source), and were harvested at intervals. For overexpression experiments, cells were harvested for analysis 48 hours after being infected with lentivirus encoding lncRNA *PTSL*, *PSLR-2* or vector only. The TRIzol (Ambion) method and company instructions were used to isolate total RNA; however, the isopropanol incubation was extended overnight at −20°C to aid in RNA precipitation. Following conversion of RNA to cDNA with the SuperScript III First-Strand Synthesis System (Invitrogen), SYBR Green qPCR Mastertmix (Qiagen) was used to measure levels of lncRNA and mRNA, in triplicate. C_T_ values were first normalized to *β-ACTIN* and then plotted as 2^−Δ^*^C^*^t^, 2^−ΔΔ^*^C^*^t^, or log_2_ fold-change (see Figure Legends for details). Primer sequences for *PTSL* are forward 5′-GAGTGAGACAGTGGGCTTGA and reverse 5′-CAATGGATTCCTGCCTTTGCC. Primers for other genes/lncRNA were published previously (35). For RNA-seq, A549 cells were harvested for analysis 48 hours after being infected with lentivirus encoding lncRNA *PTSL*, *PSLR-2* or vector only (quadruplicate samples). GENEWIZ/Azenta performed the RNA-seq library preparation (NEBNext Ultra II RNA Library Preparation Kit) and generated raw RNA-seq profiles (Illumina HiSeq 4000). After obtaining the results, the data were analyzed as described above.

### Cell Growth Assay

Sixteen hours following lentiviral infection of A549, H460, H1299, or PC9 cells to express lncRNA *PTSL, PSLR-2*, or *MALAT1* or vector only, 2,500 cells/well (quadruplicates) were placed into 96-well plates. Following an 8-hour incubation to allow the cells to adhere, MTT proliferation assays (Sigma) were performed, as per manufacturer's instructions, to measure the 0 hour reading (562 nm) and at 24 hours intervals to evaluate cell growth over time.

### Colony Formation Assay

After lentiviral infection to express lncRNA *PTSL, PSLR-2*, or *MALAT1* or vector only, low-density cultures of A549 and H460 (200 cells/well, 6-well plates, in triplicate) were cultured for 2 weeks. Crystal violet (0.5% in methanol) was then used to stain the colonies. Colonies (≥50 cells) were counted using a dissecting microscope and representative pictures were taken (LG-G6 phone with 13MP standard-angle lens).

### Cell Number, Viability, and Cell-cycle Evaluation

LUAD cells expressing lncRNA *PTSL, PSLR-2*, or *MALAT1* or vector only (see above), were placed into 6-well plates (1 × 10^5^ cells/well). Trypan Blue dye exclusion assays were used to determine live cell number and viability after 48 hours; the mean of four independent experiments with one sample each for each cell line was quantified. For cell-cycle analysis, one sample per condition per timepoint for each cell line was harvested, propidium iodide was allowed to intercalate into DNA, and flow cytometry performed to evaluate the cell-cycle profile. The percentage of DNA in each phase was quantified using the Dean-Jett-Fox model on FlowJo (BD; RRID:SCR_008520); four independent experiments were performed for both cell lines. To detect cells in mitosis, intracellular staining for phosphorylated Histone H3 (phospho-S10; Abcam ab267372; RRID:AB_2934071) was performed (two independent experiments) according to the manufacturer's instructions. Colcemid-treated cells (16 hours at 0.05 μg/mL; Gibco) were included for a positive control for cells arrested in M-phase.

### Western Blotting

A549 and H460 cells expressing lncRNA *PTSL, PSLR-2*, or vector only (see above) were placed in 10 cm plates (5 × 10^5^). After 48 hours, cells were harvested and whole-cell protein lysates were prepared using RIPA lysis buffer (50 mmol/L Tris pH 7.4, 150 mmol/L NaCl, 1% sodium deoxycholate, 1% Triton X-100, 0.1% SDS). Equal amounts of protein per sample were separated by SDS-PAGE, transferred to nitrocellulose membranes, and proteins Western blotted. Antibody details published previously (35).

### LUAD Patient Samples

Deidentified surgical resections of patients with non–small cell lung adenocarcinoma and normal lung tissue were obtained following patient consent from the lung biorepository at Vanderbilt University Medical Center (Nashville, TN). Samples were evaluated by a board-certified pathologist using hematoxylin and eosin–stained sections to confirm that the tumor samples contained >80% tumor cells and that the normal tissue had no indication of precancerous/cancerous lesions.

### Statistical Analysis

For the RNA-seq data that contain read counts or normalized values, we performed edgeR (32) or unpaired *t* tests, respectively, to identify differentially expressed genes/lncRNAs. To correct for the occurrence of false positives by chance, we computed the FDR using the BH method (34). For the biological experiments (e.g., MTT, colony, cell number, cell cycle, and qRT-PCR), which required comparing two groups, we used unpaired *t* tests. To calculate combined *P* values, we used one-tailed statistical tests as required by Fisher method. The remaining tests were two-tailed. We indicated the name of specific statistical tests used for individual analyses in the text and/or figure/table legends. We considered statistical tests with *P* values of ≤0.05 as significant. However, if a more stringent *P*-value cutoff was used, it is in the text and/or figure/table legends.

### Bioinformatics Data Availability

The RNA-seq data generated in this study were deposited in the GEO database (accession id: GSE217068). All publicly available data used in this study can be obtained from ChIPBase (http://rna.sysu.edu.cn/chipbase/), Depmap (https://depmap.org/portal), GEO (https://www.ncbi.nlm.nih.gov/gds/), Tanric (https://bioinformatics.mdanderson.org/public-software/tanric/), TCGA (https://portal.gdc.cancer.gov/), Xena (https://xena.ucsc.edu/cite-us), and WebGestalt (http://www.webgestalt.org/). Details on the publicly available RNA-seq and ChIP-seq data analyzed in this study are in Supplementary Table S1. The R code used for our computational pipeline is available on GitHub (https://github.com/compbio78/Core_p53_effector_lncRNAs.git).

## Results

### Multi-omics Integration Reveals Pan-tumor Suppressive Core p53-regulated lncRNAs

**FIGURE 1:**
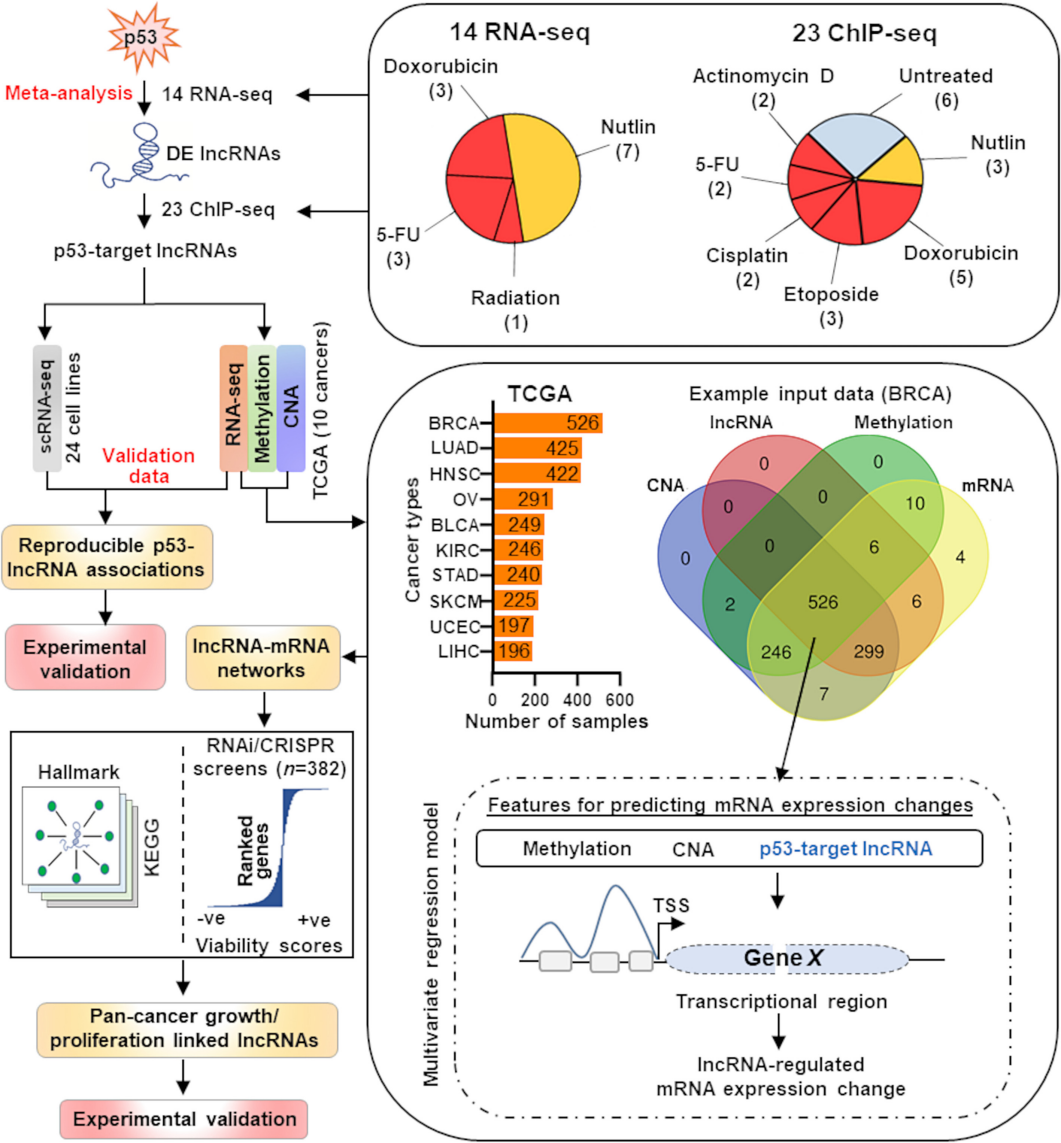
Integrated computational and experimental framework for identifying novel p53-regulated tumor-suppressive lncRNAs. Diagram of an integrated computational and experimental workflow for predicting p53-effector lncRNAs whose transcriptional upregulation by p53 activation would predict a reduction in cancer cell survival/growth. Using meta-analysis of the indicated number of RNA-seq and ChIP-seq datasets in p53-activated cells (top-right), potential direct target lncRNAs of p53 were determined. As independent validation data, scRNA-seq of cell lines and bulk RNA-seq of tumor samples were analyzed to verify consistency in p53-effector lncRNA expression. A multivariate regression function was modeled to predict p53-effector lncRNA-mediated mRNA expression changes in individual cancer types after controlling for the impact of DNA methylation and copy-number alteration (CNA) on gene expression (bottom-right). The process was repeated for 10 different TCGA cancer types shown in the bar graph. Next, an integrative approach predicted whether individual lncRNA-associated genes, derived from the above multivariate regression model, were enriched with cancer pathways/hallmark gene sets, or cell survival/growth-associated genes (bottom-left). Identification of pan-cancer proliferation/growth suppressive lncRNAs and experimental validation of the p53-dependent tumor-suppressive lncRNAs identified.

We developed an integrative bioinformatics framework to elucidate TFs regulating core target lncRNAs, which are thought to be primarily cell/tissue-specific, across cell types and under different conditions and the functions of these lncRNAs (Fig. 1). We used p53 as our model TF to decode core p53-effector lncRNAs that are consistently transactivated by and that mediate the tumor-suppressive effects of p53 across cell types and stresses. Our framework started with the evaluation of 14 high-quality RNA-seq and 23 ChIP-seq datasets from different cell types with wild-type p53 (p53^WT^) treated with diverse p53-activating agents (Fig. 1; Supplementary Table S1). To verify the results, we evaluated the core p53-effector lncRNA levels in independent bulk (*n* = 10 TCGA cancer types) and single-cell (*n* = 24 cancer cell lines) RNA-seq (scRNA-seq) data across cancer types where cells/tissues have p53^WT^ or p53 loss-of-function (p53^LOF^) mutations/truncating alterations, or the cells were treated with either p53-activating stimuli or vehicle control, respectively. Our multivariate statistical model utilized the genomic, epigenetic, and transcriptomic profiles of 3,017 patient samples across 10 different cancer types available in TCGA (Fig. 1; Supplementary Table S2) to identify high-confidence lncRNA-mRNA networks. To address whether core p53-effector lncRNAs mediate tumor-suppressive activities of p53 across cancers, we developed an approach that integrated lncRNA-mRNA networks and CRISPR and/or short hairpin (sh)/siRNA-mediated genome-scale loss-of-function screens across 382 cancer cell lines of the same 10 cancer types (refs. 41–43; Fig. 1; Supplementary Table S3). Our approach, utilizing these large-scale resources, provided us an unprecedented ability to distinguish pan-cancer regulating core p53-target lncRNAs from whole lncRNAome data.

### Meta-analysis Reveals p53-transactivated lncRNAs Across Diverse Stresses

**FIGURE 2:**
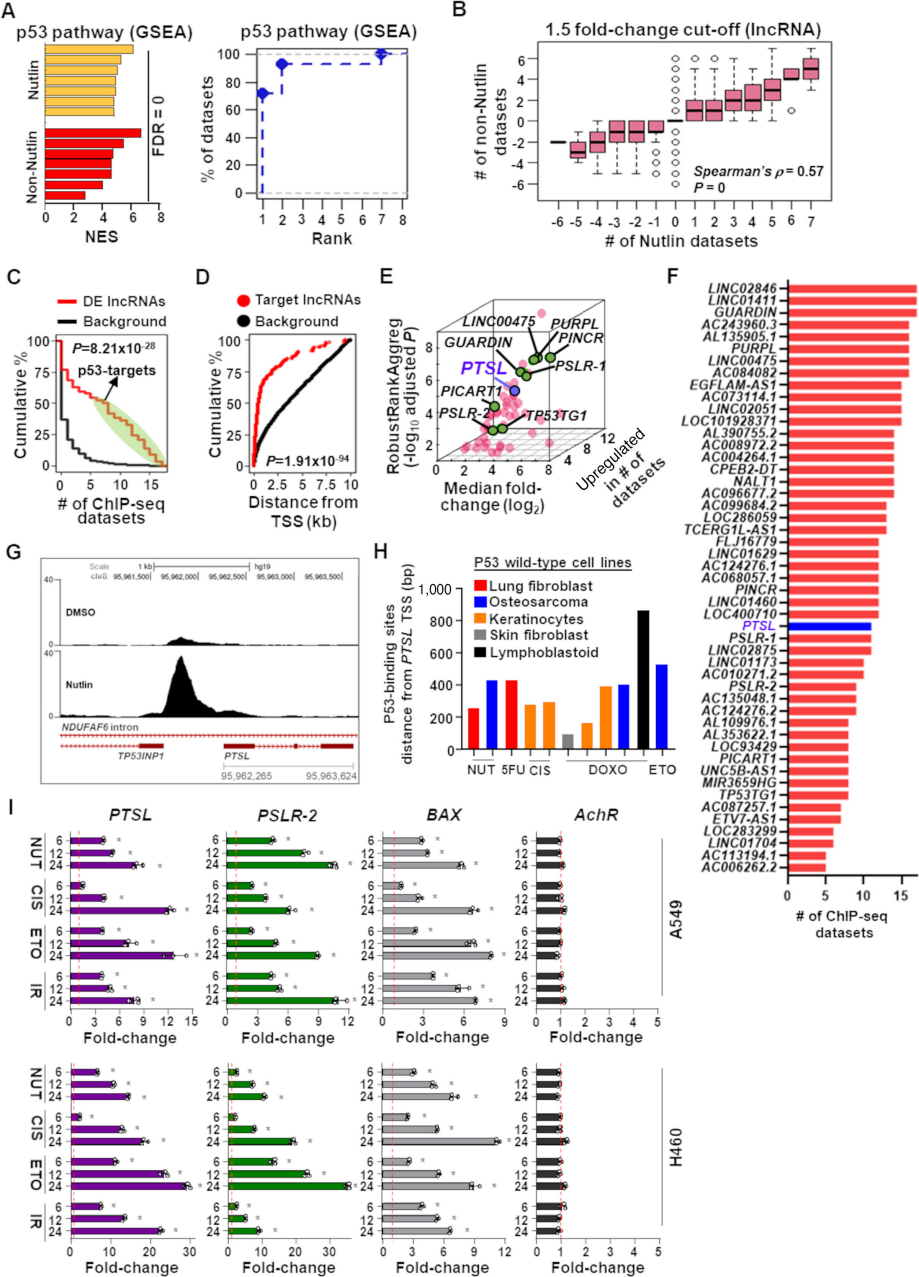
Meta-analysis to identify consistent p53-transactivated lncRNAs across cell types. **A,** Enrichment of p53 pathway in 14 RNA-seq datasets following p53 activation. Plots (bar graph left, empirical cumulative distribution right) indicate whether the p53 pathway was significantly elevated following treatment with different p53-activating agents by providing normalized enrichment scores (NESs), significance, and ranks. GSEA, gene set enrichment analysis; FDR, false discovery rate. **B,** Box-plot with Spearman rank correlation statistics indicate whether lncRNA expression changes have concordance between the cells treated with Nutlin and non-Nutlin agents. Expression changes (log_2_ fold-change) of lncRNA expression determined by comparing cells treated with p53-activating agents to those treated with vehicle control. **C,** Number of ChIP-seq datasets showing p53 binding to the indicated percentage of meta-analysis derived differentially expressed (DE) lncRNAs or background lncRNAs. **D,** Distribution of p53-binding sites around the transcription start site (TSS) of potential p53-target lncRNAs and the background lncRNAs. *P* value indicates significance in the difference of the distributions (Wilcoxon rank-sum test; C and D). **E,** Distribution of median fold-change, adjusted RobustRankAggreg significance levels, and number of datasets with elevated expression of p53-effector lncRNAs. Green and red circles indicate known and previously unreported p53-regulated lncRNAs, respectively. The blue circle denotes the previously unreported p53-regulated lncRNA experimentally verified by this study. **F,** ChIP-seq datasets showing lncRNAs bound by p53. *x*-axis indicates the number of ChIP-seq studies that determined p53-binding sites in the promoter region of the lncRNA listed on the *y*-axis. **G,** Evaluation of two representative p53-ChIP-seq data from Sammons and colleagues (74). IMR90 cells (Supplementary Table S1) demonstrate differential peaks of lung fibroblast cells treated with p53-activating agent Nutlin compared with DMSO vehicle control. Chromosome locations denoted. **H,** Consistent p53-binding sites within 900 bp upstream of the *PTSL* transcription start site (*y*-axis) in 11 p53-ChIP-seq datasets from five different p53 wild-type cell types following treatment with one of the five p53-activating agents (*x*-axis; NUT: Nutlin, 5FU: 5-Fluorouracil, CIS: Cisplatin, DOXO: Doxorubicin, ETO: Etoposide); lung fibroblast: IMR90; osteosarcoma: U2OS; skin fibroblast: GM06170, GM00011; and lymphoblastoid: GM06993, GM11992. **I,** qRT-PCR (triplicate) for *PTSL* and controls of two LUAD cell lines (A549 and H460) treated with 10 μmol/L of Nutlin (NUT), cisplatin (CIS), or etoposide (ETO), or 5 Gy gamma radiation (IR) at 6, 12, and 24 hours. RNA levels normalized to *β-ACTIN* and values plotted as 2^−ΔΔ^*^C^*^t^, with vehicle control–treated cells set at 1 (red line); data are mean ±SD. For A549, *PTSL* *, *P* < 3.35 × 10^−4^, *PSLR-2* *, *P* < 2.51 × 10^−5^, and *BAX* *, *P* < 2.93 × 10^−4^; for H460, *PTSL* *, *P* < 1.9 × 10^−6^, *PSLR-2* *, *P* < 3.52 × 10^−3^, and *BAX* *, *P* < 1.21 × 10^−5^.

We ranked mRNAs and lncRNAs based on their differential expression fold-changes following p53 activation with one of four different stimuli compared with controls in each of the 14 RNA-seq datasets, representing eight different cell types (Fig. 1; Supplementary Table S1). Seven datasets were from cells treated with Nutlin-3 (Nutlin) or a Nutlin derivative (Idasanutlin/RG7388), which blocks p53 from binding its negative regulator, MDM2, causing p53 stabilization and activation (47) (Nutlin group; Supplementary Table S1). The remaining seven datasets were from cells treated with doxorubicin, 5-fluorouracil, or gamma radiation, which are DNA-damaging agents/stimuli that activate p53 (non-Nutlin group; Supplementary Table S1). The ranked genes were subjected to GSEA. We determined the p53 pathway was significantly (FDR = 0) upregulated in all 14 datasets with 13 ranking this pathway first or second (Fig. 2A). These data show the p53 pathway was activated in these datasets and possessed the expected transcriptome-wide changes, indicating they are qualitatively good for the identification of p53-effector lncRNAs.

High concordance in lncRNA and mRNA differential expression patterns were observed in Nutlin and non-Nutlin treated datasets and was consistent across different expression fold-change cut-off values (range of Spearman *ρ *=0.45–0.85 with correlation *P* = 0; Fig. 2B; Supplementary Fig. S1). Considering the concordance in the transcriptomic changes following treatment with p53-activating agents, we employed the RobustRankAggreg (v1.1) method (33) to conduct a meta-analysis across all 14 diverse RNA-seq datasets to determine the mRNAs and lncRNAs that were consistently upregulated or downregulated with statistical significance (FDR ≤ 0.05; Materials and Methods section). In addition, we analyzed 23 p53 ChIP-seq datasets, representing multiple different cell types (Fig. 1; Supplementary Table S1). We identified lncRNAs with p53-binding sites within 10 kb of their transcription start sites. We determined the average number of p53-bound lncRNAs in Nutlin (*n* = 1,080) or non-Nutlin (*n* = 983) treated cells was >2.6-fold higher compared with untreated/vehicle control cells (*n* = 375). A significantly (*P =* 8.21 × 10^−28^; Wilcoxon rank-sum test) higher number of ChIP-seq datasets had p53-binding sites in the meta-analysis–derived significantly differentially expressed lncRNAs than the remaining lncRNAs (background data) annotated by the GENCODE database (version 27; ref. 9; Fig. 2C). We considered a lncRNA to be a potential direct p53 target if it had p53-binding site(s) in five or more ChIP-seq datasets and was bound by p53 following treatment with Nutlin and one or more non-Nutlin p53–activating stimuli (Fig. 2C). From the combined RNA-seq and ChIP-seq analyses, we identified 50 high-confidence p53-regulated lncRNAs and of those, 49 were upregulated upon p53 activation (Supplementary Table S4), suggesting that, similar to protein-coding genes, direct binding of p53 to lncRNA promoters preferentially results in their transcriptional activation instead of repression (6, 7, 48). Considering the most proximal p53-binding site relative to the transcription start site in each ChIP-seq dataset, we determined p53 had significantly (*P =* 1.91 × 10^−94^; Wilcoxon rank-sum test) closer proximity to these 49 potential p53-target lncRNAs compared with the remaining annotated lncRNAs (Fig. 2D). Also, the expression of these lncRNAs was elevated in a greater number of p53-activated RNA-seq datasets (median datasets = 9; median fold-change = ∼2–225; Fig. 2E; Supplementary Table S4) and bound by p53 in a higher number of ChIP-seq datasets (*n* = 5–17, median = 12; Fig. 2F), suggesting they may be core p53-effector lncRNAs.

It was recently reported that following p53 activation, 380 altered lncRNAs were identified in liver HepG2 cells (49). We determined that compared with background lncRNAs, a significantly (*P* = 1.27 × 10^−31^; hypergeometric test) higher proportion of our predicted p53-effector lncRNAs overlapped with these altered lncRNAs, increasing confidence that our identified lncRNAs are core p53 targets. We also conducted a literature search for lncRNAs directly regulated by p53 and it revealed that among the 49 potential p53-effector lncRNAs we identified, eight [*GUARDIN/LNCTAM34A* (20), *LINC00475* (17), *PICART1* (22), *PINCR* (13), *PURPL* (19), and *TP53TG1* (21)], including the two we previously identified [*PSLR-1* and *PSLR-2* (35)] have been reported to be p53 targets (Fig. 2E, green circles). Our analysis of approximately 16,000 annotated lncRNAs (9) indicated that only 49 were identified as core p53-regulated lncRNAs, which supports the belief that lncRNA expression is highly cell-type specific. However, the 49 lncRNAs transactivated by p53 across different cell types and stresses determined by our meta-analysis indicate the existence of core p53-regulated lncRNA.

Among the 49 predicted p53-target lncRNAs, the majority were annotated as long intergenic non-coding RNAs (lincRNA, *n* = 28) or antisense RNAs (*n* = 14) in GENCODE (version 27; Supplementary Table S4). We focused on a previously unknown p53-effector lncRNA ENSG00000253878 [also called *RP11-347C18.3* or *AC087752.3*, annotated in LNCipedia (50)], hereafter referred to as *PTSL* (p53-regulated tumor-suppressive lncRNA). Subcellular localization information from multiple cell lines in the lncAtlas database indicates *PTSL* is likely to be nuclear (51). *PTSL* is considered to be an alternatively spliced [predicted by VastDB (52)] sense-intronic lncRNA. It is encoded on chromosome 8 within a non–protein-coding intronic region of *NDUFAF6* that is 73.6 kb upstream from the protein-coding region of *NDUFAF6*. There is a polyadenylation signal sequence (AAUAAA) present at the 3′ end of *PTSL*, suggesting it is likely polyadenylated. In addition, *PTSL* appears to share a promoter with *TP53INP1*, a known p53-target protein-coding gene (53) encoded on the opposite strand and in the opposite direction from *PTSL* (Fig. 2G). Following p53 activation, 11 of 17 ChIP-seq datasets revealed p53-binding sites immediately upstream (<900 bp) of the transcription start site of *PTSL* (Fig. 2G and H). Furthermore, *PTSL* was consistently elevated in 12 of 14 RNA-seq datasets following p53 activation (median fold-change = 4.7; FDR = 1.99 × 10^−4^; Fig. 2E; Supplementary Table S4). Of note, *TP53INP1* was also significantly elevated (median fold-change = 4.7; FDR = 3.43 × 10^−5^) with p53 activation across the RNA-seq datasets, but *NDUFAF6* was not (median fold-change = 1.31; FDR = 1). These data indicate that although *PTSL* is encoded in an intronic region of *NDUFAF6*, this gene is not regulated by p53 as *PTSL* and *TP53INP1* are.

To further validate the meta-analysis results, we experimentally evaluated *PTSL* levels in two p53^WT^ LUAD cell lines (A549 and H460) after treatment with Nutlin, cisplatin, etoposide, or gamma radiation to activate p53. Compared with vehicle control–treated cells, expression of *PTSL* was significantly increased following treatment with any of the four p53-activating stimuli (Fig. 2I). The lncRNA *PSLR-*2, a known p53 target lncRNA, and *BAX*, a well-known p53 target gene, were evaluated as positive controls. The gene encoding the acetyl choline receptor (*AchR*) was a negative control since it is not regulated by p53. Therefore, we validated the meta-analysis that p53 activation results in increased expression of *PTSL*, and also showed this occurs in another cancer type (lung cancer) not included in the 14 RNA-seq datasets (Supplementary Table S1). Altogether, our results indicate the identified high-confidence p53-target lncRNAs are induced upon p53 activation across different cell types.

### Validation of p53-effector lncRNA Alterations Across Diverse Cancers at a Single Cell Level

**FIGURE 3:**
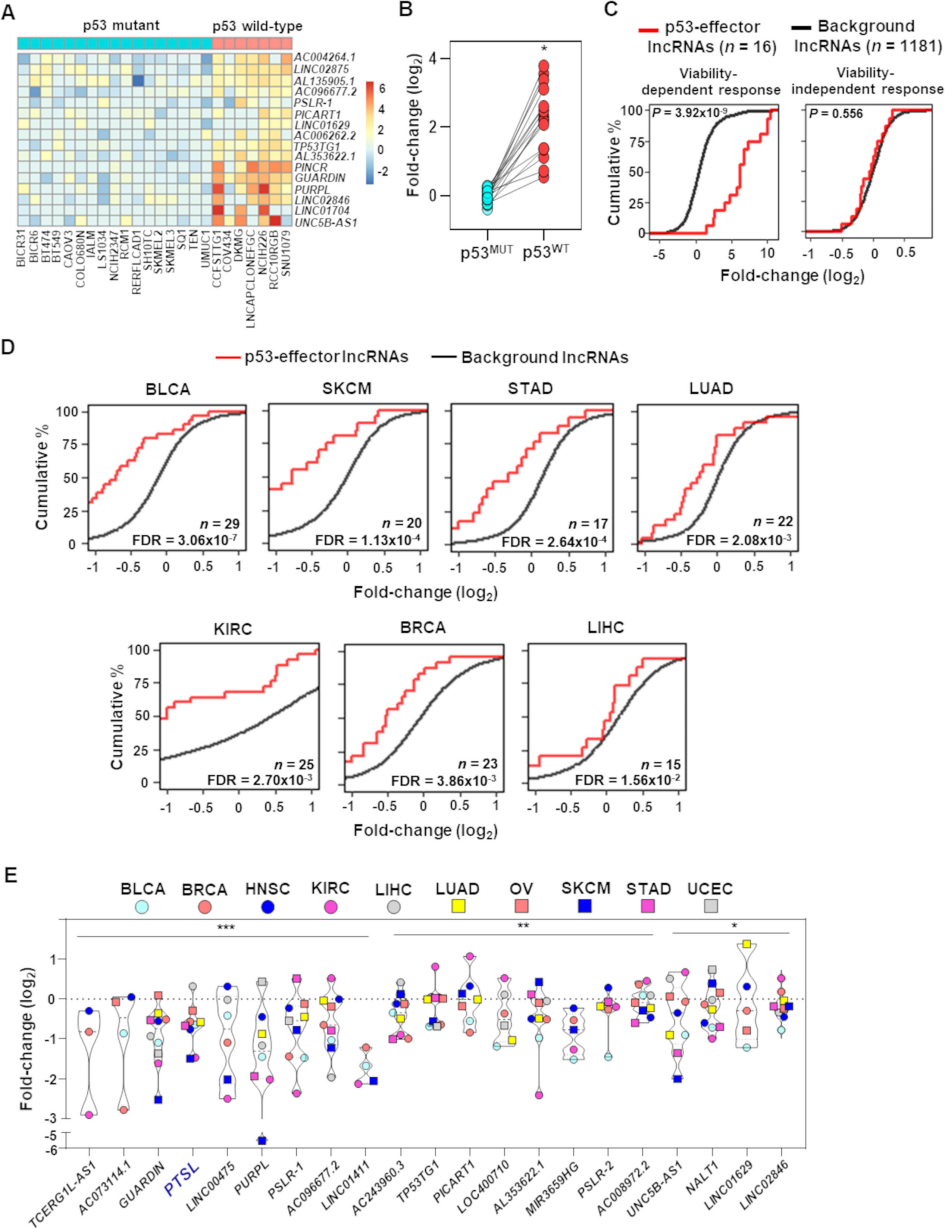
Functional p53 is necessary to regulate core p53-effector lncRNA expression. **A,** Heatmap showing fold-change (log_2_) of the indicated p53-effector lncRNAs in the indicated idasanutlin-treated cell line compared with vehicle control [scRNA-seq from McFarland et al. (54)]. **B,** Distribution of the expression fold-changes (log_2_) of the lncRNAs in p53^MUT^ and p53^WT^ cell lines from A; *, *P =* 1.33 × 10^−7^, *t* test. **C,** Empirical cumulative distribution of expression fold-changes (log_2_) of p53-target lncRNAs (*n* = 16; indicated in A) and background lncRNAs (*n* = 1,181) for the idasanutlin-sensitive (viability-dependent response, left) and idasanutlin-insensitive (viability-independent response, right) components. *P* values from Wilcoxon rank-sum test. **D,** Empirical cumulative distribution of p53-effector and background lncRNA expression changes in TCGA samples with p53 inactivating mutations compared with wild-type p53. Total p53-effector lncRNAs detected in a specific cancer type is denoted by *n*. *y*-axis estimates percentage of lncRNAs at or below a log_2_ fold-change value indicated on *x*-axis. *P*-values from Wilcoxon rank-sum test. **E,** Meta-analysis of p53-effector lncRNA expression changes in samples with p53^LOF^ compared with p53^WT^ in 10 different cancer types (TCGA). *, *P <* 0.05; **, *P <* 9.5 × 10^−6^; ***, *P <* 5.6 × 10^−17^, FDR-corrected Fisher combined probability test.

Utilizing recently published massively multiplexed scRNA-seq datasets, we evaluated lncRNA expression following treatment with p53-activating idasanutlin (Nutlin-3 derivative) in 24 diverse cancer cell lines (54). Many lncRNAs were not captured by single-cell technology due to poor detection of non-polyadenylated transcripts (55), high dropout rates, and lower expression of lncRNAs compared with mRNAs. Despite these limitations, among the detected lncRNAs, we were able to assess whether treatment results in higher expression changes of the p53-regulated lncRNAs compared with non–p53-regulated (background) lncRNAs. We determined that our identified p53-target lncRNAs were more highly expressed in idasanutlin-treated p53^WT^ cells compared with vehicle control–treated cells (Fig. 3A). Notably, this was not evident in cells harboring mutations (in-frame deletion, missense and nonsense mutations, splice site variants) in p53 (p53^MUT^, Fig. 3A, ref. 56). Also, the distribution of lncRNA expression fold-changes in p53^WT^ cells was significantly (*P =* 1.33 × 10^−7^; *t* test) higher compared with p53^MUT^ cells (Fig. 3B). The scRNA-seq data provide additional support that the identified potential core p53-target lncRNAs are regulated in a p53-dependent manner across multiple cancers.

Using a statistical modeling approach described previously (54), we next estimated the association between lncRNA expression changes in cell lines and the viability of the cells in response to idasanutlin treatment, from the drug sensitivity database GDSC (57) and PRISM (58, 59). This method categorizes the expression change of each lncRNA into a viability-independent response component that characterizes the lncRNA expression changes in idasanutlin insensitive cell lines and a viability-related response component that characterizes the expression difference between idasanutlin sensitive and insensitive cell lines. We determined the p53-effector lncRNAs detected have a significantly higher fold-change (*P* = 3.92 × 10^−9^; Wilcoxon rank-sum test) compared with the background lncRNAs with a viability-related/dependent response, but not with a viability-independent response (Fig. 3C). These results indicate our identified p53-effector lncRNAs are preferentially more sensitive to p53 activation from idasanutlin compared with the remaining lncRNAs.

### p53 Inactivation Reduces Expression of Core Target lncRNAs Across Cancer Types

With the analysis of 3,017 patient samples from 10 different TCGA cancer types (Fig. 1), we investigated whether the expression of our meta-analysis derived lncRNAs is reduced in samples with p53 loss-of-function (p53^LOF^) and whether it is consistently observed across multiple cancer types. For each cancer type, we divided the samples into p53^WT^ and p53^LOF^ (nonsense mutation, frameshift insertion/deletion, and splice site variants; Supplementary Table S2). We determined that seven of 10 cancer types with p53^LOF^ had reduced expression of a significantly (FDR < 0.05; Wilcoxon rank-sum test) higher percentage of p53-regulated lncRNAs compared with the percentage of background lncRNAs (Fig. 3D); a similar trend was observed in an eighth cancer type (Supplementary Fig. S2). We also determined that 21 of the 49 identified p53-effector lncRNAs were recurrently downregulated with statistical significance (FDR-corrected Fisher combined *P <* 0.05) across the cancer types with p53^LOF^ (Fig. 3E). Notably, the lncRNA *PTSL*, which had significantly elevated levels upon p53 activation across the RNA-seq and our qRT-PCR analyses, showed recurrent downregulation with p53 loss across TCGA cancer types with statistical significance (FDR-corrected Fisher combined *P* = 1 × 10^−18^). The lncRNAs that show p53-dependent alteration across cancers and stresses suggest they have pan-tumor suppressive functions.

### Core p53-target lncRNAs Negatively Regulate Pan-cancer Oncogenic Processes

We next investigated whether expression of the 49 p53-targeted lncRNAs may negatively impact critical tumorigenic processes. For the same 10 TCGA cancer types, we first identified lncRNA-associated genes in each cancer, using a multivariate regression model (Fig. 1). The model captured only those genes whose expression were predicted to be modulated with the expression of a lncRNA, accounting for the impact of copy-number changes and DNA methylation, which modulate gene expression. From the predicted lncRNA-gene pairs, we conducted a comprehensive enrichment analysis to identify which biological pathways and cancer Hallmark gene sets were significantly changed across the cancer types with the alteration of core p53-target lncRNAs. For each lncRNA, we identified the top 200 significantly (FDR-corrected regression *P* < 10^−3^) negatively and top 200 significantly positively associated genes. To determine which pathways in the Kyoto Encyclopedia of Genes and Genome (KEGG; ref. 60) database were significantly (FDR-corrected hypergeometric test *P* < 0.05) enriched with the individual lncRNA-associated genes, we identified those pathways that were preferentially upregulated or downregulated by p53-effector lncRNAs across 10 TCGA cancer types. The pathway Cell cycle that induces proliferation was downregulated, while the tumor-suppressive pathway p53 was upregulated by a higher number of p53-effector lncRNAs across the cancer types than the number of lncRNAs that had the opposite impact (Supplementary Fig. S3; Supplementary Tables S5 and S6).

**FIGURE 4:**
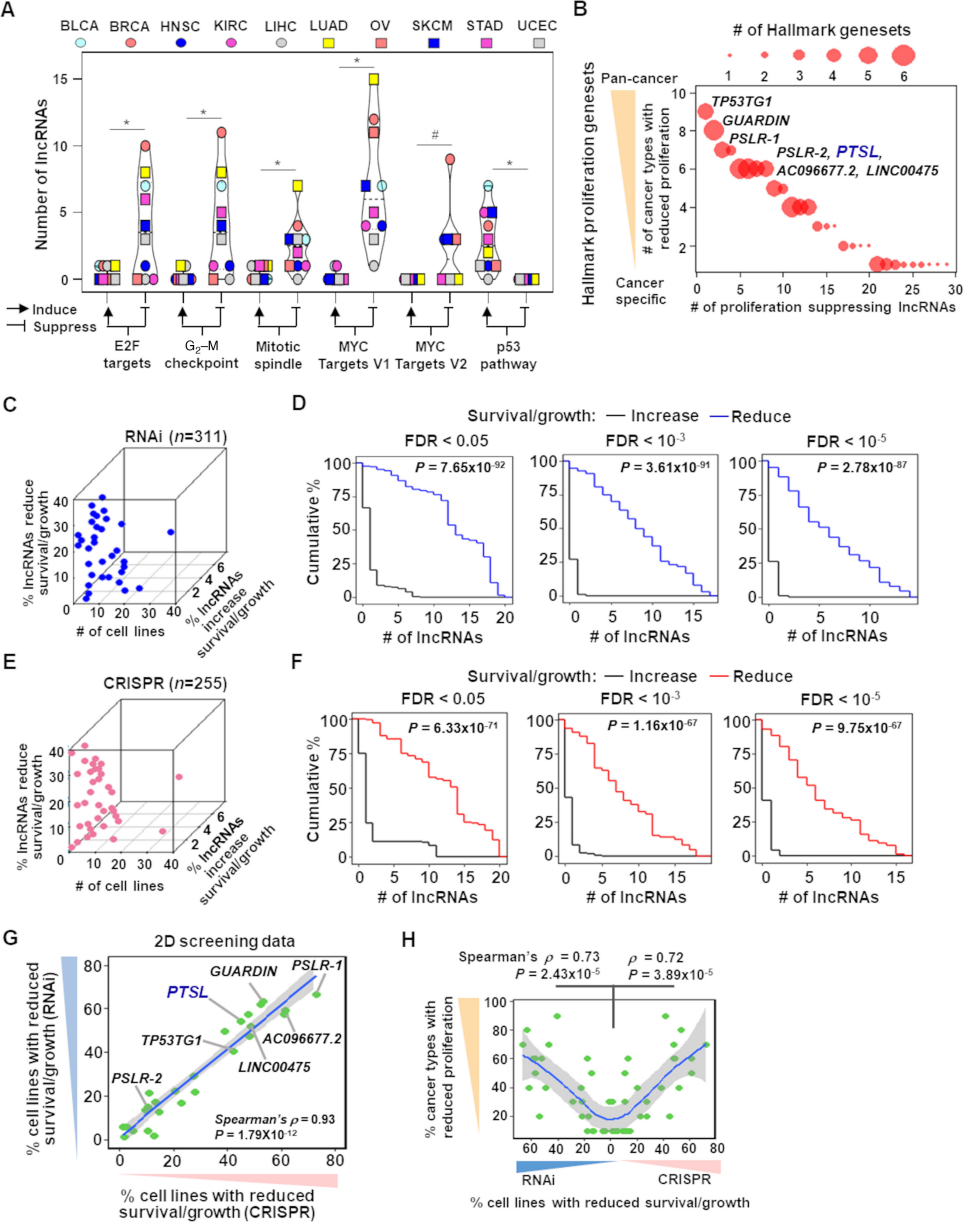
Core p53-target lncRNAs negatively regulate pan-cancer cell growth. **A,** The number of p53-effector lncRNAs that potentially induce/suppress individual Hallmark gene sets linked with cell proliferation. Significant differential distribution, FDR corrected *, *P* < 0.038; ^#^, *P* = 0.063; Wilcoxon rank-sum tests. **B,** Bubble-plot shows the number of p53-effector lncRNAs (*x*-axis) that potentially suppress the indicated number (dot size) of proliferation-linked processes in how many cancers (*y*-axis). Top prioritized lncRNAs are labeled. **C–F,** 3D plots illustrate the percent of p53-effector lncRNAs that potentially reduce (*y*-axis) or induce (*z*-axis) survival/growth in the number of cell lines (*x*-axis) in RNAi (C) or CRISPR (E) screening data; *n* = number of cell lines analyzed. Empirical cumulative distribution of the number of lncRNAs that potentially increase or reduce survival/growth in individual cancer cell lines from RNAi (D) and CRISPR (F) screening data at three different FDR (FDR represents adjusted gene set enrichment *P* value) thresholds. *y*-axis estimates percentage of cell lines linked with at or below the lncRNA number indicated in *x*-axis. *P* values from Wilcoxon rank-sum test. **G,** Scatterplots with Spearman rank correlations indicate consistency in the expression of p53-effector lncRNAs and their negative impact on cancer cell survival/growth in two independent screening datasets [*x*- (CRISPR) and *y*- (RNAi) axes]. **H,** Scatterplots with Spearman rank correlations indicate consistency in p53-effector lncRNAs expression and their negative impact on tumor proliferation in patient samples across cancer types (*y*-axis) and cell survival/growth measured in cancer cell lines using RNAi (*x*-axis; left) or CRISPR (*x*-axis; right).

For each p53-effector lncRNA, the positively or negatively associated genes were selected to investigate which cancer Hallmark gene signatures (61) were significantly (FDR < 0.05) enriched (Supplementary Tables S7 and S8). We first sorted all 50 Hallmark gene signatures into eight broad categories. Distribution of p53-effector lncRNAs across these categories of Hallmark processes (61) indicated a significantly higher number of lncRNAs negatively impacted/suppressed cancer cell proliferation (4-fold higher; FDR *=* 2.03 × 10^−4^; Wilcoxon rank-sum test) in cancer cells compared with the number of lncRNAs that positively impacted/induced the same processes (Supplementary Fig. S4). Further investigation of individual gene sets linked to proliferation determined that a significantly (FDR < 0.038; Wilcoxon rank-sum test) higher number of lncRNAs potentially suppressed the oncogenic gene sets (E2F targets, G_2_–M checkpoint, Mitotic spindle, MYC targets) compared with the number of lncRNAs that potentially induced the same processes (Fig. 4A). In contrast, there was a significantly increased (FDR = 1.17 × 10^−2^; Wilcoxon rank-sum test) number of lncRNAs that induced the tumor-suppressive p53 pathway (Fig. 4A). Altogether, the results indicate that the identified core p53-effector lncRNAs may critically contribute to the pan-tumor suppressive functions of p53. In support of this, Hallmark data analysis determined that core p53-effector lncRNAs predominantly suppress proliferation (*P* = 1.16 × 10^−9^; Wilcoxon rank-sum test; Supplementary Fig. S5). Notably, *PTSL* and six other p53 transactivated lncRNAs (*TP53TG1*, *GUARDIN*, *PSLR-1*, *PSLR-2*, *AC096677.2*, and *LINC00475*) potentially suppress proliferation in >5 cancer types by modulating ≥5 proliferation-associated Hallmark gene sets (ref. 61; Fig. 4B). Collectively, these analyses suggest the p53-effector lncRNAs we identified possess pan-tumor proliferation suppressive activity.

### CRISPR/RNAi Screens Reveal Pan-tumor Growth Suppressive lncRNAs

We further investigated whether the activation of meta-analysis–derived p53-effector lncRNAs potentially reduce cancer cell survival/growth, which would consequently lead to tumor suppression. We utilized genome-scale CRISPR and/or sh/siRNA based loss-of-function screens for 382 cancer cell lines originating from the same 10 TCGA cancer types evaluated above (41–43). Here, a gene with a lower score indicates the cell has a greater dependency on that gene for survival/growth. First, for each cancer type, the p53-effector lncRNA-associated mRNAs were determined from the multivariate regression analysis (Fig. 1). For the cancer cell lines originating from this cancer type, we used GSEA (44) to determine the lncRNAs with significantly (FDR < 0.001) enriched associations with genes whose inactivation reduced cancer cell survival/growth. We divided these lncRNAs into potentially oncogenic or tumor-suppressive groups based on whether they induce or suppress the expression of survival/growth-promoting genes, respectively. This analysis revealed that individual cell lines had a significantly (*P* < 1.2 × 10^−67^; Wilcoxon rank-sum test) higher number of p53-regulated lncRNAs whose overexpression potentially reduces cell survival/growth compared with those that potentially increase cell survival/growth (Fig. 4C–F; Supplementary Tables S9–S12). Preferential tumor-suppressive activities of p53-effector lncRNAs were consistent across different GSEA significance levels (FDR < 0.05, <10^−3^, and <10^−5^; Fig. 4D and F). The survival/growth associations between the lncRNAs and the number of cell lines were consistent in RNAi and CRISPR screening data (Spearman *ρ *= 0.93; correlation *P* = 1.79 × 10^−12^; Fig. 4G). Employing the same approach to analyze three-dimensional (3D) spheroid CRISPR screening data of LUAD cell lines (H1975, H2009, H23) showed that p53-effector lncRNAs, including *PTSL*, potentially negatively impact LUAD cell survival/growth (Supplementary Fig. S6). In addition, the lncRNAs that potentially suppress proliferation in a larger number of TCGA cancer types also had a negative impact on cell survival/growth in a larger number of cancer cell lines (Spearman *ρ* ≥0.72, *P* < 4 × 10^−5^; Fig. 4H).

The seven lncRNAs, indicated in Fig. 4B, that were prioritized as recurrent proliferation suppressors across TCGA cancers showed suppression of cell survival/growth across the cancer cell lines in both two-dimensional (2D; 13%–73%; median = 53% of the cell lines in both CRISPR and RNAi screening data) and 3D screening data (Fig. 4G and Supplementary Fig. S6, respectively). These lncRNAs were significantly (FDR-corrected Fisher combined *P <* 10^−5^) downregulated with p53^LOF^ recurrently across TCGA cancer types (Fig. 3E) and upregulated with p53 activation across multiple RNA-seq datasets (Supplementary Table S4), indicating p53 likely regulates their expression. Previous reports show *TP53TG1*, *GUARDIN*, and *LINC00475* are directly regulated by p53 in one or two cancer types (17, 20, 21). We previously verified p53-dependent expression alteration and tumor-suppressive functions of *PSLR-1* and *PSLR-2* (35). The two other lncRNAs, *PTSL* and *AC096677.2*, were not previously reported to be p53 targets or to have tumor-suppressive functions. Collectively, our integrative bioinformatics data identified p53-regulated lncRNAs whose elevated expression suggest they suppress tumorigenesis in multiple cancer types, including those that are aggressive, such as LUAD, and indicate they are pan-tumor growth suppressive.

### PTSL has a Tumor-suppressive Function That Positively Impacts Cancer Patient Survival

**FIGURE 5:**
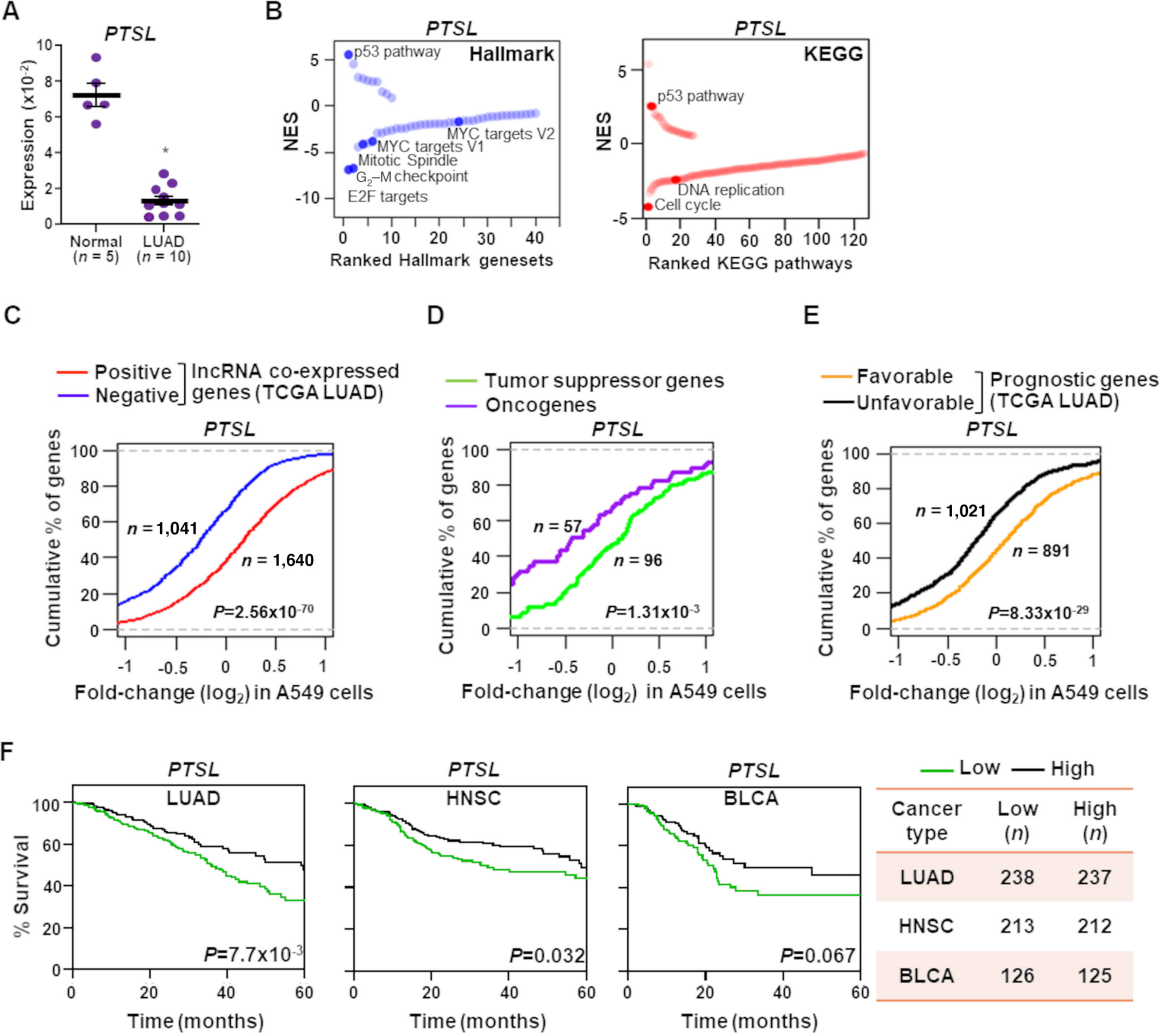
*PTSL* expression is associated with tumor-suppressive functions and increased cancer patient survival. **A,** qRT-PCR (triplicate) for *PTSL* expression in normal human lung tissue (*n* = 5) and LUAD patient samples (*n* = 10). Values were normalized to *β-ACTIN* and presented as 2^−Δ^*^C^*^t^; data are mean ±SEM; *, *P* = 1.22 × 10^−7^. **B,** RNA-seq after expressing *PTSL* or vector control in A549 LUAD cell line (quadruplicate). GSEA showed top upregulated and downregulated Hallmark gene sets (left) or KEGG pathways (right) in A549 cells with *PTSL* overexpression compared with vector control. Upregulated and downregulated gene sets/pathways were indicated by positive and negative normalized enrichment score (NES), respectively. Proliferation-associated gene sets/pathways with significant enrichment (FDR < 0.05) are highlighted. **C,** Genes having significant (FDR-corrected regression *P* < 10^−3^) positive or negative coexpression with *PTSL* in TCGA LUAD data were selected to investigate their expression fold-change distribution in A549 cells expressing *PTSL* compared with vector control. **D,** Expression fold-change distribution of curated oncogenes and tumor-suppressor genes in *PTSL*-expressing A549 cells compared with vector control. **E,** Significant favorable and unfavorable prognostic genes in TCGA LUAD patient cohort were selected. Expression fold-change distribution of these genes in *PTSL*-expressing A549 cells compared with vector control. For C–E, gene expression fold-changes shown on the *x*-axis, *y*-axis represents percentages of genes below the log_2_ fold-change value indicated in the *x*-axis. *P* values from Wilcoxon rank-sum test. *n* denotes number of genes. **F,** Overall survival associations (Kaplan–Meier plots) between lncRNA *PTSL* and the patients of the indicated cancer types. Using median cutoff of *PTSL* expression, we divided the patient samples into low and high groups. Using log-rank tests, we measured significantly different survival outcomes; *n* denotes sample number.

To validate our bioinformatic data, we evaluated the expression of the previously unreported core p53-regulated tumor-suppressive lncRNA *PTSL* in our own LUAD patient samples. *PTSL* levels were significantly (*P* = 1.22 × 10^−7^; *t* test) lower in LUAD patient samples compared with normal lung tissue (Fig. 5A). To begin to experimentally validate the potential growth-suppressive function of *PTSL* in cancer cells, independent of potential p53-inducing drug effects, we engineered A549 LUAD cells to express *PTSL* or vector control. Following RNA-seq to measure transcriptome-wide expression changes, we performed GSEA (44) and identified which Hallmark gene sets or KEGG (60) pathways were significantly (FDR < 0.05) altered with elevated levels of *PTSL*. We determined the p53 pathway was among the top significantly upregulated pathways, which is consistent in both Hallmark gene sets (FDR = 0) and KEGG pathways (FDR = 1 × 10^−3^) in A549 cells with elevated levels of *PTSL* (Fig. 5B). In contrast, the proliferation-linked Hallmark gene sets [E2F targets (FDR = 0), G_2_–M checkpoint (FDR = 0), Mitotic spindle (FDR = 0), and MYC targets V1 (FDR = 0) and V2 (FDR = 0.045)] and KEGG pathways [Cell cycle (FDR = 0) and DNA replication (FDR = 3 × 10^−3^)] were in the top downregulated gene sets/pathways (Fig. 5B), suggesting that *PTSL* may have a negative impact on these processes. These results are consistent with those obtained from TCGA pan-cancer data analysis (Fig. 4A), indicating growth-suppressive functions of the lncRNA *PTSL*.

Besides the consistency observed at the pathway level between TCGA LUAD and our LUAD RNA-seq data, we also evaluated reproducibility in the gene-level association. We determined genes with significant positive association (FDR-corrected regression *P* < 0.001) with *PTSL* in TCGA LUAD patient data had significantly higher expression (*P* = 2.56 × 10^−70^; Wilcoxon rank-sum test) in the *PTSL* perturbed LUAD RNA-seq data compared with those that had significant negative associations (Fig. 5C). Moreover, we evaluated expression of literature-curated high-confidence tumor suppressors (62) and oncogenes (63) that have significant downregulation and upregulation (≥2 fold-change with FDR < 0.05), respectively, in TCGA LUAD patient samples. We determined a significantly (*P* = 1.31 × 10^−3^; Wilcoxon rank-sum test) distinct distribution of tumor suppressors and oncogenes, where tumor-suppressor genes showed preferential upregulation and oncogenes showed preferential downregulation with *PTSL* expression (Fig. 5D). Combined results from TCGA LUAD patient data and *PTSL* perturbation data in LUAD cells revealed that *PTSL* likely suppresses tumor growth by suppressing oncogenic processes while inducing p53-mediated tumor-suppressive processes.

To determine whether *PTSL* has an impact on cancer patient survival, we first evaluated whether expression alteration of *PTSL*-associated genes correlates with patient prognosis (Materials and Methods section). The genes showing significant (|Z-score| > 1.96 corresponds to *P* < 0.05; log-rank test; Materials and Methods section) favorable and unfavorable prognostic association with TCGA LUAD patient cohort have significantly (*P =* 8.33 × 10^−29^; Wilcoxon rank-sum test) distinct distributions in *PTSL* perturbed A549 cells where favorable and unfavorable genes were preferentially upregulated and downregulated, respectively (Fig. 5E). We next assessed whether *PTSL* expression correlates with patient survival. We determined that higher *PTSL* expression significantly correlates with increased overall survival in LUAD (*P* = 7.7 × 10^−3^; log-rank test) and head and neck squamous cell carcinoma (HNSC; *P* = 0.032), and is trending toward significant in bladder cancer (BLCA; *P* = 0.067; Fig. 5F). These data support a pan-cancer growth-suppressive function of *PTSL* that may contribute to better cancer patient prognosis.

### PTSL Inhibits Proliferation by Inducing a G_2_ Regulatory Network

**FIGURE 6:**
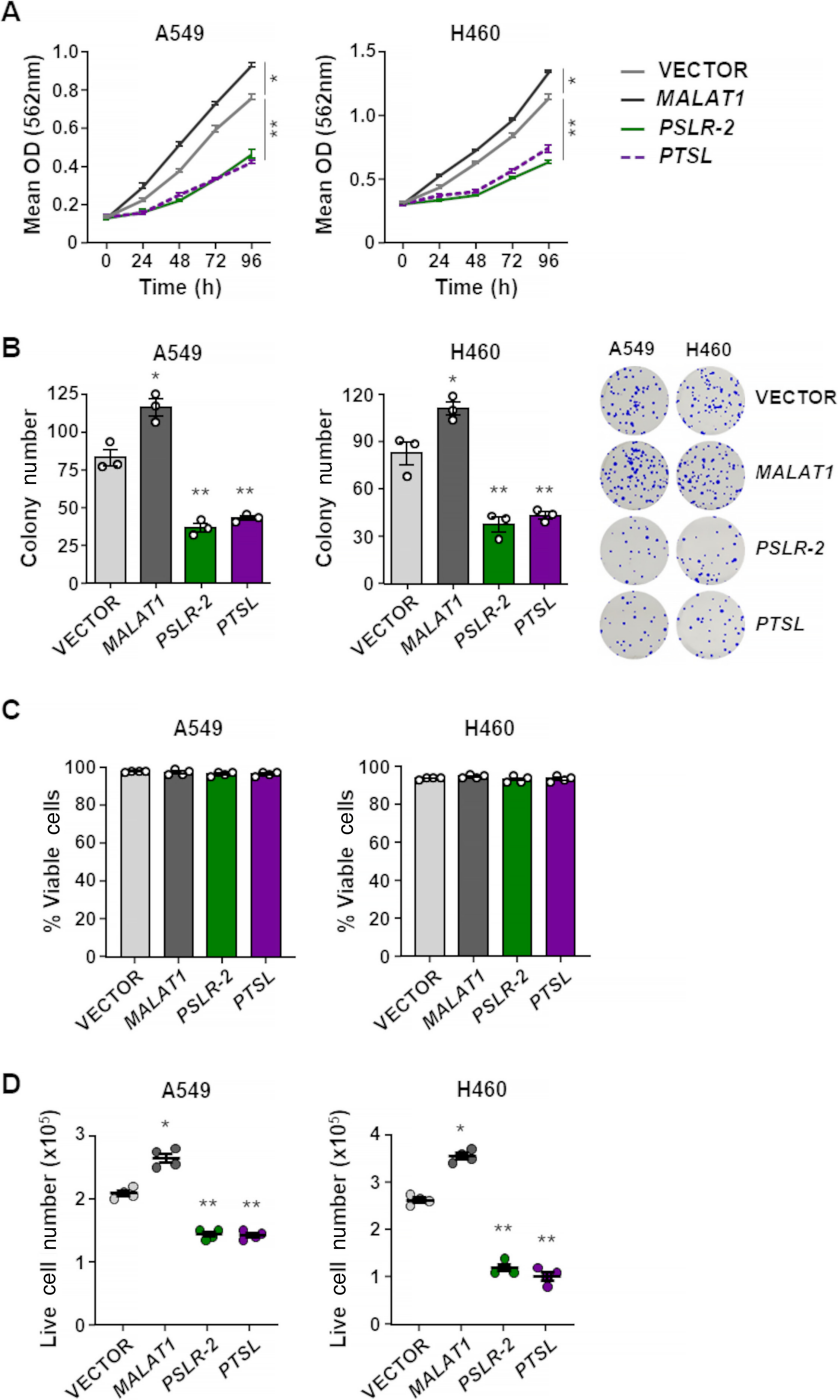
p53-regulated *PTSL* exerts tumor-suppressive activity in LUAD. The lncRNAs *PTSL*, *PSLR-2*, or *MALAT1* or vector control were expressed (lentivirus) in A549 and H460 LUAD cell lines. **A,** MTT assays (quadruplicate) performed at 24 hours intervals. Each assay was performed two to four independent times for both cell lines and one representative experiment is shown; mean ±SD; A549 *, *P* < 9.1 × 10^−4^ and **, *P* < 2.3 × 10^−3^, H460 *, *P* < 5.1 × 10^−4^ and **, *P* < 6.1 × 10^−4^. **B,** Two weeks after plating, colonies in colony formation assays were stained and quantified. Each assay was performed two to three independent times, in triplicate, for both cell lines and one representative experiment with images (no magnification) is shown; mean ±SEM; A549 *, *P* = 1.2 × 10^−2^ and **, *P* < 1.75 × 10^−3^, H460 *, *P* = 3.0 × 10^−2^ and **, *P* < 7.1 × 10^−3^. Trypan Blue dye exclusion was performed 48 hours after lentiviral infection to determine viability (**C**) and live cell number (**D**); mean of four independent experiments for both cell lines ±SD is graphed; A549 *, *P* = 5.77 × 10^−4^ and **, *P* < 2.69 × 10^−5^, H460 *, *P* = 2.9 × 10^−5^ and **, *P* < 3.63 × 10^−6^. Note, we previously reported the data for the controls (Vector, *PSLR-2*, *MALAT1*) in C and D (35), as they were evaluated at the same time the *PTSL* samples were evaluated.

Given that our data indicate *PTSL* reduces pro-proliferative pathways and thus, likely impacts cell proliferation (Fig. 5B), we further investigated this. We evaluated the biological consequences of increased levels of *PTSL* in two LUAD cell lines (A549 and H460). Compared with cells with empty vector, there was significantly reduced LUAD cell growth with increased *PTSL* expression (Fig. 6A). As lncRNA controls, we overexpressed a pro-proliferative lncRNA, *MALAT1* (64) and a growth-suppressive lncRNA *PSLR-2* (35), which expectedly increased and decreased cell growth, respectively (Fig. 6A). In addition, the number of colonies that formed from cells overexpressing *PTSL* was significantly reduced and consistent with the effects of *PSLR-2* (Fig. 6B). Although increased *PTSL* expression had no effect on LUAD viability (Fig. 6C), there was a significant reduction in live cell numbers (Fig. 6D). Similar results were observed when *PSLR-2* was expressed (Fig. 6C and D). To determine whether the growth-suppressive effects of *PTSL* require wild-type p53 to be present, we utilized H1299 (*p53*-deleted) and PC9 (p53-mutant) lung adenocarcinoma cell lines. We observed a decrease in cell growth and live cell number, but no change in viability with *PTSL* overexpression in these p53-inactivated LUAD cells (Supplementary Fig. S7A–S7C). These data indicated that *PTSL* was not inducing cell death but was impacting cell growth, and this did not require p53.

**FIGURE 7:**
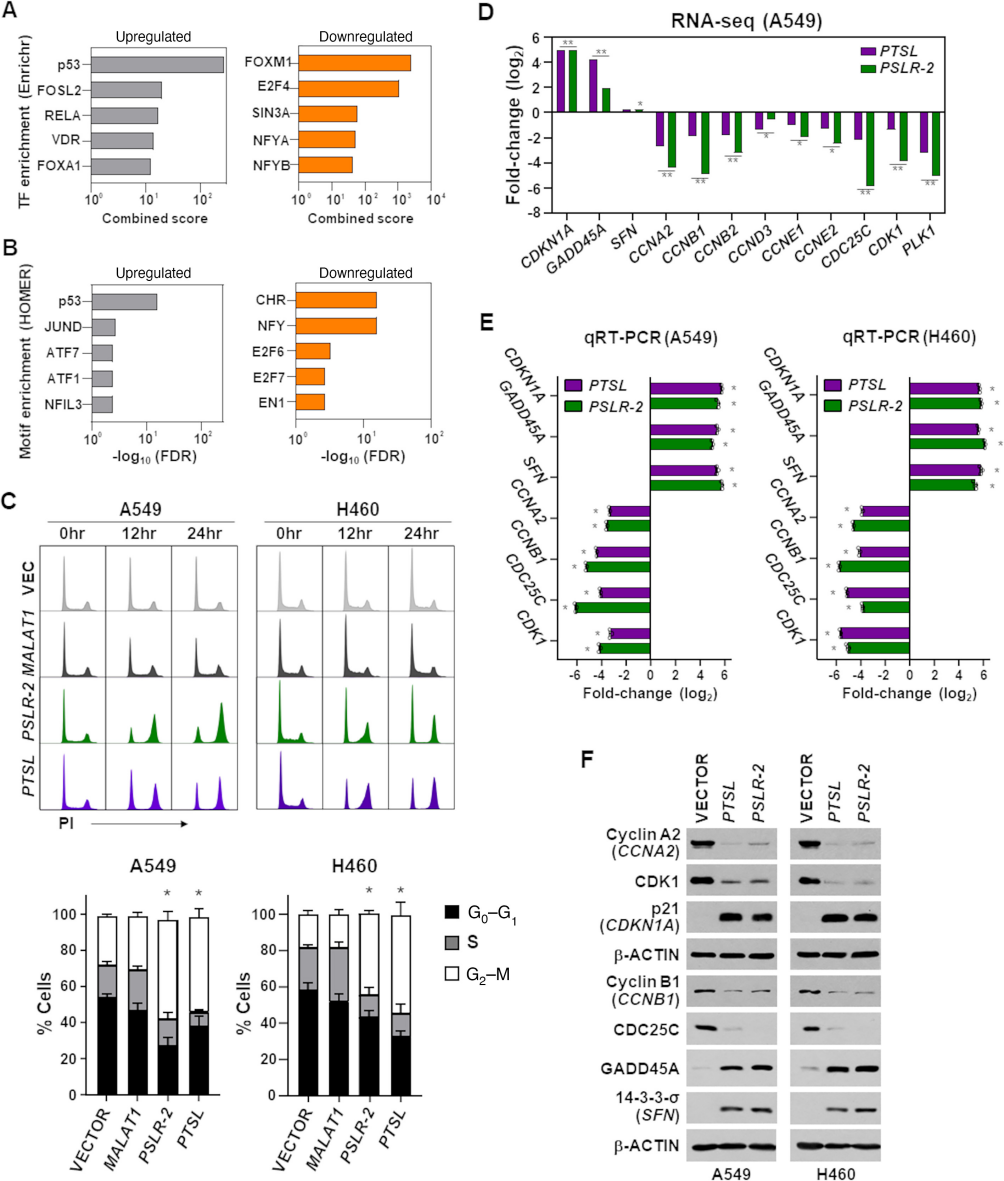
*PTSL* induces G_2_ cell-cycle arrest by activating the G_2_ regulatory network. TFs in **A** and promoter motifs in **B** showing significant (FDR < 0.05) enrichment in the genes altered upon *PTSL* expression in A549 LUAD cells. TF and motif enrichment results were obtained using Enrichr and HOMER tools, respectively. **C,** Following propidium iodide intercalation into DNA, cell-cycle analysis was performed at intervals by flow cytometry. Representative histograms (top) and quantification (bottom) of the percentage of cells in each phase as the mean of four independent experiments for both cell lines at 24 hours; ±SEM; A549 *, *P* < 1.34 × 10^−3^, H460 *, *P* < 3.42 × 10^−3^. **D,** Genes that regulate the G_2_ phase that had significantly altered expression in RNA-seq data in A549 cells with elevated *PTSL* shown. *, *P* < 6.33 × 10^−3^; **, *P* < 2.68 × 10^−15^. qRT-PCR for mRNA (**E**, triplicate) and Western blotting for protein (**F**) for the cell cycle genes indicated was performed 48 hours after lentiviral expression of *PTSL* and *PSLR-2* in LUAD cell lines. mRNA expression was normalized to *β-ACTIN* and presented as log_2_ fold-change relative to vector control; mean ±SD; A549 *, *P* < 2.15 × 10^−6^ and H460 *, *P* < 1.36 × 10^−5^.

To gain insight into the regulation of proliferation by *PTSL*, we evaluated our A549 RNA-seq data as to whether elevated *PTSL* resulted in the alteration of a random set of genes or target genes of specific TFs. We utilized the tool Enrichr (65) that integrates genome-wide ChIP-seq experiments from ENCODE (66) and ChEA (67) projects. p53 was the top enriched transcriptional regulator among the upregulated genes and FOXM1 and E2F4 were the top enriched transcriptional regulators among the downregulated genes (Fig. 7A). To increase confidence in the observed transcriptional regulation by specific TFs, we further investigated which regulatory motifs were significantly enriched in the promoter region of the genes altered by *PTSL* using the tool HOMER (68). p53 was the top enriched motif in the upregulated genes (Fig. 7B), suggesting the tumor-suppressive p53-regulatory gene networks were activated with elevated *PTSL*. In contrast, cell-cycle genes homology region (CHR) was the top enriched motif in the downregulated genes (Fig. 7B). Of note, FOXM1- and E2F4-regulated cell-cycle gene promoters are enriched with the DNA element CHR (69). The FOXM1 containing protein complex activates G_2_–M cell-cycle genes with CHR sites (69, 70). These results suggest that *PTSL* expression may lead to growth suppression through G_2_–M cell-cycle arrest.

We next directly assessed the cell-cycle effects following expression of *PTSL* in two p53 wild-type expressing LUAD cell lines and detected increased numbers of cells in the G_2_–M-phase of the cell cycle (Fig. 7C). Similarly, expression of *PTSL* in H1299 (*p53*-deleted) and PC9 (p53-mutant) cells also arrested cells in G_2_ (Supplementary Fig. S7D). To determine whether LUAD cells were stalling in G_2_- or M-phases, phospho-Histone H3, a marker of mitosis (71) was evaluated. There was a similar percentage of *PTSL*-expressing LUAD cells with phospho-Histone H3 as those with vector control (Supplementary Fig. S7E). Thus, these cells were arresting in G_2_ and not M and were analogous to the results of the growth-suppressing lncRNA *PSLR-2*, which arrests cells in G_2_ (35). These data demonstrate that *PTSL* exerts its tumor-suppressive function by inducing a G_2_ cell-cycle arrest, decreasing cell proliferation, and this did not require functional p53.

To further investigate *PTSL*-induced G_2_ cell-cycle arrest, we determined from our RNA-seq data which G_2_-regulating genes had significant alterations following increased expression of *PTSL* (Fig. 7D; ref. 72). We determined that with elevated *PTSL* levels there was increased expression (median fold-change = 19.14; median FDR = 1.93 × 10^−48^) of three genes and reduced expression of nine genes (median fold-change = 3.51; median FDR = 2.42 × 10^−20^), and this would result in a G_2_-phase cell-cycle arrest. qRT-PCR of seven of the G_2_-linked cell-cycle regulator genes in two LUAD lines expressing *PTSL* or vector control validated the changes in expression of these genes detected in RNA-seq. *CDKN1A* (p21), *GADD45A*, and *SFN* (14-3-3-σ) were significantly elevated, whereas *CCNA2*, *CCNB1*, *CDC25C*, and *CDK1* were significantly downregulated in both LUAD lines (Fig. 7E). In addition, changes in the protein levels of these genes in *PTSL*-expressing LUAD cells were analogous to the expression changes observed at the RNA level and reflect their function in G_2_ (Fig. 7F). However, levels of p53 protein were unchanged with *PTSL* expression (Supplementary Fig. S7F). Therefore, the expression of the G_2_ cell-cycle arrest network is coordinately altered by *PTSL*, demonstrating its p53-induced tumor-suppressive functions.

## Discussion

The p53 tumor suppressor is the most studied TF with a total of over 110,000 PubMed entries. It is well known that p53 transcriptionally induces protein-coding genes across cell types and regulates cellular processes to inhibit tumorigenesis by blocking cell-cycle progression, protecting genome stability, and promoting apoptosis (1, 48). Over decades, significant effort has been made toward identifying a core set of transcriptional targets of p53 that induce its tumor-suppressive functions across cell/tissue types and stresses, but this has not been fully borne out (1, 6, 7, 73). Recently, similar to protein-coding genes, p53-mediated transcription of lncRNAs was shown (8), and multiple p53-regulated lncRNAs (e.g., *Loc285194/TUSC7*, *NEAT1*, *PICART1*, *ST7-AS1*, *TP53TG1*) were reported to have tumor-suppressive functions in one or two cancer types (21–26). Because most lncRNAs are considered to be cell-type specific, it was thought to be unlikely that there would be pan-cancer lncRNAs that would mediate the effects of p53 across many different malignancies. However, in this study, we developed a bioinformatics framework that integrated large-scale high-quality p53-activated ChIP-seq and RNA-seq expression profiles with pan-cancer multilayered omics profiles to identify high-confidence core p53-target lncRNAs and their functions in tumorigenesis. These p53-regulated lncRNAs had recurrent overexpression with p53 activation in multiple cell types and across different stresses and recurrent downregulation with p53^LOF^ mutations in patient samples across cancer types. These core p53-target lncRNAs had not previously been reported and their regulation by p53 was unknown. Furthermore, unlike other meta-analyses, our study was not limited to only determining high-confidence gene targets of p53. Rather, our extended data analysis using genomic, epigenetic, and transcriptomic profiles integrated into our bioinformatics framework determined potential functional consequences of the p53-effector lncRNAs in hundreds of cancer cells and thousands of patient samples across 10 different cancer types. Our pan-cancer analyses indicate that the identified core p53-target lncRNAs are downregulated in multiple cancer types due to their regulation of core processes, specifically G_2_ cell-cycle arrest, that inhibit tumor promotion and cancer cell growth. Thus, our work provides a significant leap forward in understanding critical non–protein-coding p53 targets and the important contributions p53-regulated lncRNAs have in mediating tumor suppressor functions across cell types.

Using two different approaches, we established links between tumorigenesis and the lncRNAs that are predicted to be directly transcriptionally induced by p53 in various cell types and by different stresses, which added significant power to our analysis. First, we conducted a rigorous enrichment analysis across 10 cancer types to identify associations between p53-effector lncRNAs and the gene sets that are linked with cancer pathways. We determined a subset of the previously unreported p53-effector lncRNAs have a recurrent negative impact on proliferation across cancer types. Such reproducible/consistent results for a primary p53-regulated cellular process (1) strongly indicate true biological events, and our LUAD cell results validated this. Second, we utilized genome-scale loss-of-function screens to determine the p53-effector lncRNAs that have a negative impact on cell viability/growth across hundreds of cancer cell lines originating from the same 10 cancer types. We observed that lncRNA and cancer cell survival associations are strongly reproducible in two independent 2D screening datasets, RNAi and CRISPR. In addition, 3D CRISPR screens on lung cancer cell lines further strengthened our prediction results. Among the lncRNAs that have pan-cancer downregulation potentially due to p53 inactivation, seven showed their downregulation may induce pan-cancer cell survival/growth across the high-throughput data analyses. Taken together, we identified p53-effector lncRNAs whose downregulation in cancers, likely due to p53 inactivation, consequently favors malignant cell growth and/or survival.

Recent meta-analysis indicated that approximately 40% of the reportedly direct p53-activated genes were not identified in any of the 16 genome-wide studies of p53 target genes that covered a broad range of cell types and stresses (6). Thus, a critical objective of our large-scale data analysis was to minimize false-positives in every step to achieve high-confidence results across cell types. Therefore, we first conducted a meta-analysis on 14 RNA-seq datasets and selected only those lncRNAs that had consistent upregulation or downregulation with adjusted statistical significance in at least two-thirds of the datasets where they were detected. Second, we selected only those lncRNAs as potential direct p53 targets if they had p53-binding sites in their promoter regions in at least five ChIP-seq datasets. We identified 49 lncRNAs that were potentially activated and only one lncRNA that was potentially repressed by p53, respectively. This result is in agreement with the current belief that p53 is primarily a transcriptional activator rather than repressor (6, 7, 48). Although eight of our 49 predicted p53-effector lncRNAs are known p53 targets (13, 17, 19–22, 35), providing confidence in our meta-analysis results, there were a few known p53 lncRNA targets in human cells not identified by our approach. Specifically, *NEAT1* and *PANDA* were not significantly (FDR < 0.05) altered in our meta-analysis, and *DINO* and *lincRNA-p21* were not in GENCODE version 27, the annotation database we utilized. Although not all previously reported p53-regulated lncRNA met our stringent approach or were absent in the universally used databases utilized, we identified a set of 49 core p53-effector lncRNAs that were consistently altered by p53 across many diverse cancer types and showed these non-coding RNAs facilitate the tumor suppressive effects of p53.

To reduce the effect of DNA methylation and copy-number changes in predicting lncRNA-mediated mRNA expression changes, we conducted a multivariate regression analysis accounting for the effect of genetic and epigenetic events on gene expression. Our pan-cancer data analysis determined that a subset of the identified lncRNAs recurrently suppress oncogenic functions across cancer types and were recurrently downregulated with p53 inactivation. Such recurrent associations across cancer types that include genetic data showing p53 functional associations demonstrate reproducible/consistent results. We also evaluated independent RNAi and CRISPR screens to identify reproducible lncRNAs and cell viability/growth associations, which highly likely minimize false-positive results. For example, in our study, the p53-regulated onco-lncRNA *PURPL* (19) was overexpressed across the RNA-seq data; however, it was not in our predicted pan-tumor suppressive lncRNA group, indicating our integrative approach is robust to exclude tumor-promoting targets. Altogether, we took care in every step of our data analysis to reduce false-positives in our prediction results and obtained highly confident p53-targeted lncRNAs with tumor-suppressive functions.

We determined that levels of *PTSL* increase following treatment with a variety of p53-activating agents that are used in cancer treatments (1) both in multiple large-scale datasets and in LUAD cell lines. Increased *PTSL* expression was associated with reduced cell survival/growth in RNAi and CRISPR screens, and we experimentally validated this in p53 wild-type LUAD cells, which showed G_2_ cell-cycle arrest with increased levels of *PTSL*. Because analogous results were obtained with p53 mutant and deleted *PTSL*-overexpressing LUAD cells, the cell-cycle arrest effects of *PTSL* did not require p53. However, we cannot exclude the possibility that in the absence of p53, *PTSL* cooperated with a p53 family member. Importantly, our study provides clinical relevance by revealing that an elevated level of *PTSL* results in favorable overall patient survival in multiple cancer types.

Our experimental observations were supported by our RNA-seq data and further validation experiments, demonstrating that expression of key G_2_-linked cell-cycle regulatory genes were significantly altered at both the RNA and protein levels by expression of *PTSL*. Moreover, from the perturbation profiles of *PTSL*, we determined its elevated expression suppressed pro-proliferative pathways while inducing the tumor-suppressive p53 pathway, and preferentially suppressed the oncogenic and unfavorable prognostic genes while inducing the tumor-suppressive and favorable prognostic genes, confirming the role of *PTSL* in tumor suppression. Although *PTSL* does not appear to alter p53 levels, it is possible, because p53 was the top enriched TF motif we identified when *PTSL* was expressed, that *PTSL* facilitates p53-target gene expression directly or indirectly. Additional investigations are needed to determine the precise mechanism of *PTSL* effects. Altogether, our results indicate that induction of *PTSL* in cancer cells through stress-inducing stimuli results in reduction of cell growth, which may consequently prolong overall patient survival.

Collectively, our integrative bioinformatics framework has exposed a group of lncRNAs in the highly cell/tissue type-specific lncRNAome, which were transactivated by p53 across different cell types and stresses that mediate the tumor-suppressive functions of p53 in diverse cancer types. Our multiple lines of experimental validation demonstrated that the proposed framework is robust in identifying high-quality, critical core p53 target lncRNAs and importantly, this approach can be applied to other transcriptional regulators in cancer or other diseases.

## Supplementary Material

Supplementary Figure S1 Similarity in lncRNA and mRNA expression changes between Nutlin and non-Nutlin treated cells.

Supplementary Figure S2 Distribution of p53-regulated lncRNAs in cancers with p53LOF

Supplementary Figure S3 Expression association of individual KEGG pathways with core p53-target lncRNAs across cancer types

Supplementary Figure S4 Distribution of the number of p53-effector lncRNAs that potentially induce/suppress individual Hallmark categories

Supplementary Figure S5 Comparative results for p53-effector lncRNAs that mediate the suppression or induction of proliferation across TCGA cancer types

Supplementary Figure S6 p53-effector lncRNAs showing cell survival/growth association in 3D CRISPR screening data

Supplementary Figure S7 PTSL expression arrests LUAD cells in G2 regardless of p53 status

Supplementary Table S1 Summary of 14 RNA-seq and 23 ChIP-seq datasets evaluated

Supplementary Table S2 TCGA cancer types evaluated to identify differentially expressed lncRNAs in samples with p53LOF compared with p53WT

Supplementary Table S3 Number of cell lines of the indicated cancer types with CRISPR or RNAi screening data

Supplementary Table S4 Differential expression of 49 potential p53-effector lncRNAs across 14 RNA-seq datasets

Supplementary Table S5 KEGG pathways that have significant positive associations with p53-effector lncRNAs

Supplementary Table S6 KEGG pathways that have significant negative associations with p53-effector lncRNAs

Supplementary Table S7 Hallmark genesets that have significant positive associations with p53-effector lncRNAs

Supplementary Table S8 Hallmark genesets that have significant negative associations with p53-effector lncRNAs

Supplementary Table S9 p53-effector lncRNAs whose over-expression potentially reduce survival/growth of cancer cell lines screened by RNAi

Supplementary Table S10 p53-effector lncRNAs whose over-expression potentially increase survival/growth of cancer cell lines screened by RNAi

Supplementary Table S11 p53-effector lncRNAs whose over-expression potentially reduce survival/growth of cancer cell lines screened by CRISPR

Supplementary Table S12 p53-effector lncRNAs whose over-expression potentially increase survival/growth of cancer cell lines screened by CRISPR
