# Supplementary material for: Decoding the lncRNAome Across Diverse Cellular Stresses Reveals Core p53-effector Pan-cancer Suppressive lncRNAs
Source: Cancer Res Commun. 2023 May 11;3(5):842–59. doi: 10.1158/2767-9764.CRC-22-0473 (PMC10173889; doi:10.1158/2767-9764.CRC-22-0473)
Supplement: Supplementary Figure S1 — Similarity in lncRNA and mRNA expression changes between Nutlin and non-Nutlin treated cells. [file crc-22-0473-s01.pdf]

Supplemental Information

Supplemental Figures

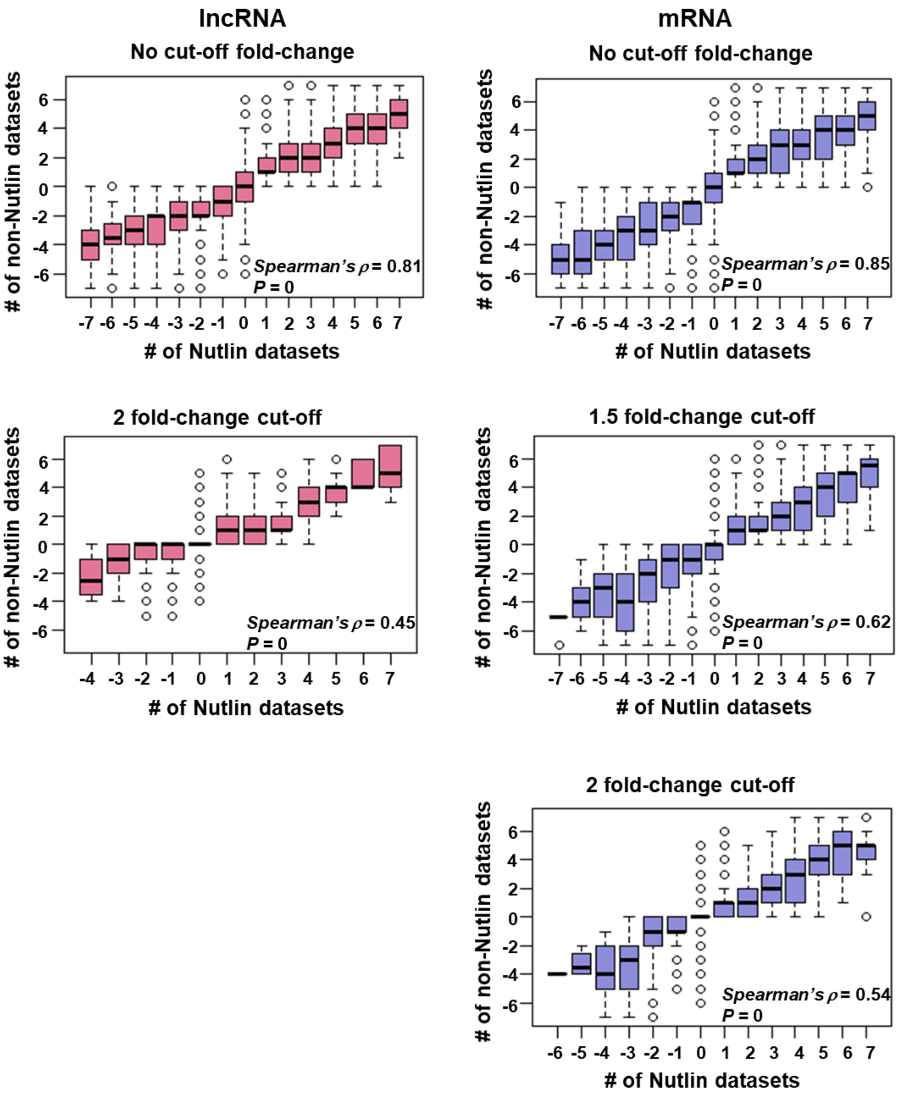

Supplementary Figure S1. Similarity in lncRNA and mRNA expression changes between Nutlin and non-Nutlin treated cells. Box-plots with Spearman's rank-correlation coefficients indicate whether lncRNA (left) and mRNA (right) expression changes in the Nutlin-treated cells have concordance with the expression changes in the cells treated by non-Nutlin agents. Expression changes (log2 fold-change) were measured from the lncRNA and mRNA expression in cells treated with p53-activating agents (Nutlin, 5-FU, doxorubicin, and gamma radiation, compared with the cells treated with vehicle control. *P*-values from Spearman's rank-correlation test.
