# Supplementary material for: Decoding the lncRNAome Across Diverse Cellular Stresses Reveals Core p53-effector Pan-cancer Suppressive lncRNAs
Source: Cancer Res Commun. 2023 May 11;3(5):842–59. doi: 10.1158/2767-9764.CRC-22-0473 (PMC10173889; doi:10.1158/2767-9764.CRC-22-0473)
Supplement: Supplementary Figure S2 — Distribution of p53-regulated lncRNAs in cancers with p53LOF [file crc-22-0473-s02.pdf]

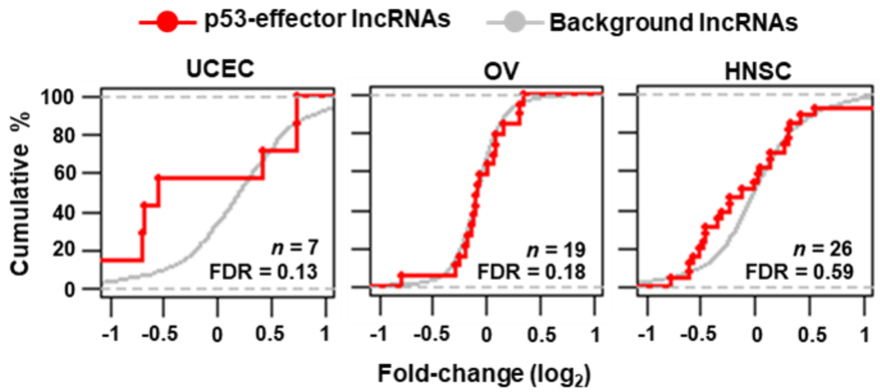

Supplementary Figure S2. Distribution of p53-regulated lncRNAs in cancers with p53^{LOF}. Empirical cumulative distribution of p53-effector and background lncRNA expression changes in samples with p53 truncating mutations compared with wild-type p53. Total p53-effector lncRNAs detected in a specific cancer type is denoted by *n*. Y-axis estimates percentage of lncRNAs at or below a log2 fold-change value indicated on x-axis. FDR corrected *P*-value was derived from two-tailed Wilcoxon-rank sum test statistic.
