# Supplementary material for: Decoding the lncRNAome Across Diverse Cellular Stresses Reveals Core p53-effector Pan-cancer Suppressive lncRNAs
Source: Cancer Res Commun. 2023 May 11;3(5):842–59. doi: 10.1158/2767-9764.CRC-22-0473 (PMC10173889; doi:10.1158/2767-9764.CRC-22-0473)
Supplement: Supplementary Figure S3 — Expression association of individual KEGG pathways with core p53-target lncRNAs across cancer types [file crc-22-0473-s03.pdf]

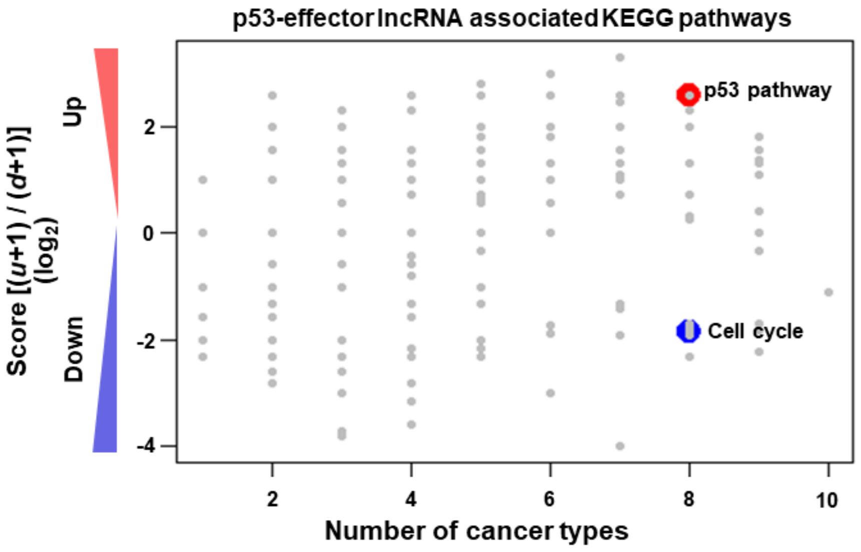

Supplementary Figure S3. Expression association of individual KEGG pathways with core p53-target lncRNAs across cancer types. Scatterplot showing individual KEGG pathways (dots) that are preferentially up- (red) or down- (blue) regulated (y-axis; scores) by the p53-effector lncRNAs across TCGA cancers (x-axis). Red/blue dots highlight specific pan-cancer up-/down-regulated pathways, respectively. *u* or *d* denotes the number of unique lncRNAs that potentially up- or down-regulated the specific pathway. Note that +1 is added to avoid infinity.
