# Supplementary material for: Decoding the lncRNAome Across Diverse Cellular Stresses Reveals Core p53-effector Pan-cancer Suppressive lncRNAs
Source: Cancer Res Commun. 2023 May 11;3(5):842–59. doi: 10.1158/2767-9764.CRC-22-0473 (PMC10173889; doi:10.1158/2767-9764.CRC-22-0473)
Supplement: Supplementary Figure S4 — Distribution of the number of p53-effector lncRNAs that potentially induce/suppress individual Hallmark categories [file crc-22-0473-s04.pdf]

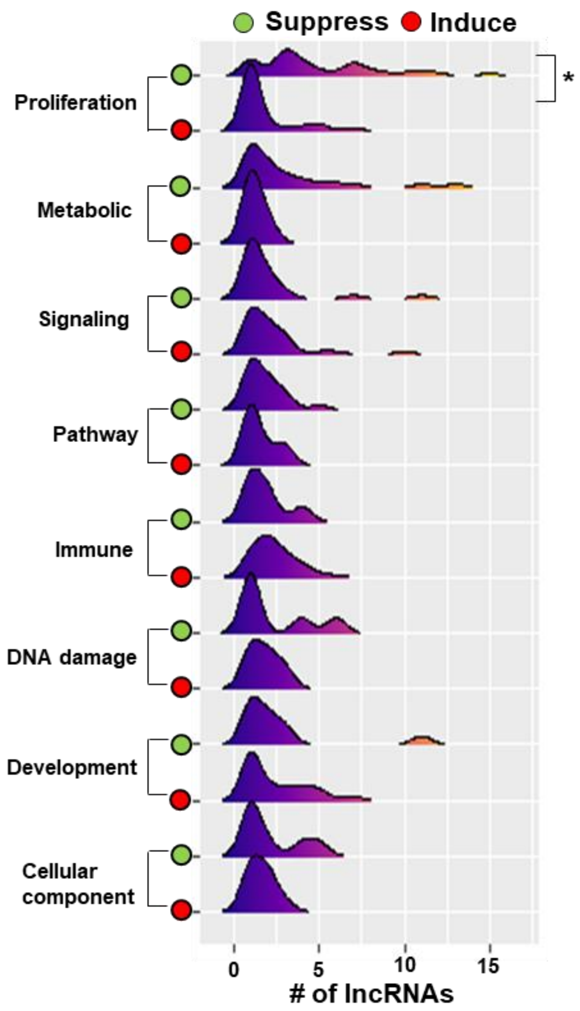

Supplementary Figure S4. Distribution of the number of p53-effector lncRNAs that potentially induce/suppress individual Hallmark categories. Each plot shows the accumulated result from 10 TCGA cancers; FDR corrected *P=2.03x10^{-4} denoting significant differential distribution.
