# Supplementary material for: Decoding the lncRNAome Across Diverse Cellular Stresses Reveals Core p53-effector Pan-cancer Suppressive lncRNAs
Source: Cancer Res Commun. 2023 May 11;3(5):842–59. doi: 10.1158/2767-9764.CRC-22-0473 (PMC10173889; doi:10.1158/2767-9764.CRC-22-0473)
Supplement: Supplementary Figure S5 — Comparative results for p53-effector lncRNAs that mediate the suppression or induction of proliferation across TCGA cancer types [file crc-22-0473-s05.pdf]

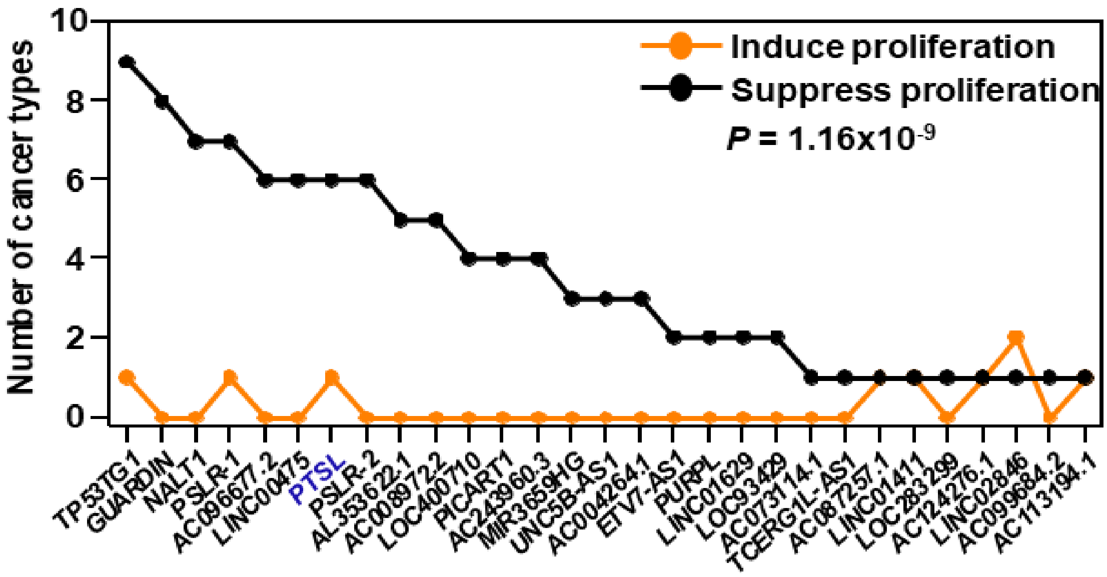

Supplementary Figure S5. Comparative results for p53-effector lncRNAs that mediate the suppression or induction of proliferation across TCGA cancer types. Dots in the lines indicate the lncRNAs (x-axis) that potentially suppress or induce proliferation-linked processes in the indicated number of cancer types (y-axis). *P*-value indicates significance in the difference of these two distributions (two-tailed Wilcoxon rank-sum test).
