# Supplementary material for: Decoding the lncRNAome Across Diverse Cellular Stresses Reveals Core p53-effector Pan-cancer Suppressive lncRNAs
Source: Cancer Res Commun. 2023 May 11;3(5):842–59. doi: 10.1158/2767-9764.CRC-22-0473 (PMC10173889; doi:10.1158/2767-9764.CRC-22-0473)
Supplement: Supplementary Figure S6 — p53-effector lncRNAs showing cell survival/growth association in 3D CRISPR screening data [file crc-22-0473-s06.pdf]

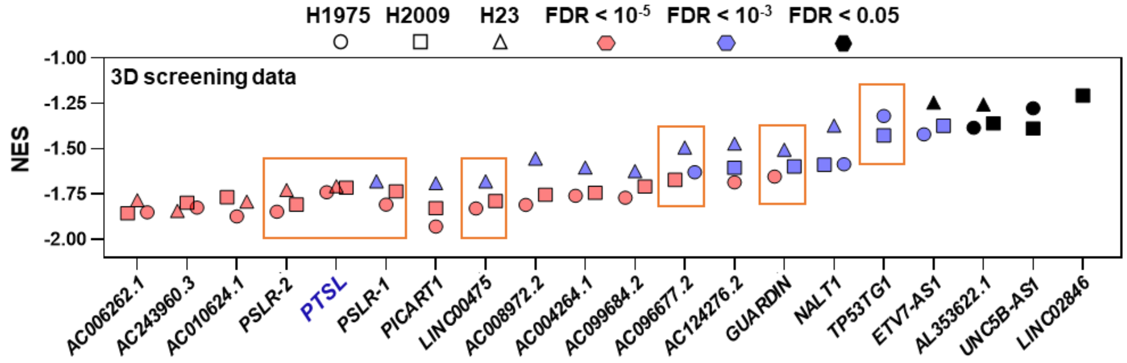

Supplementary Figure S6. p53-effector lncRNAs showing cell survival/growth association in 3D CRISPR screening data. Gene Set Enrichment Analysis (GSEA) based on 3D CRISPR screening data shows lncRNAs that potentially negatively impacted cell survival/growth of LUAD cell lines. Normalized enrichment scores (NES) indicate the strength of the negative impact. Colors indicate different statistical significance levels (FDR, false discovery rate). Orange boxes identify top prioritized lncRNAs highlighted in Figures 4B and 4G.
