# Supplementary material for: Decoding the lncRNAome Across Diverse Cellular Stresses Reveals Core p53-effector Pan-cancer Suppressive lncRNAs
Source: Cancer Res Commun. 2023 May 11;3(5):842–59. doi: 10.1158/2767-9764.CRC-22-0473 (PMC10173889; doi:10.1158/2767-9764.CRC-22-0473)
Supplement: Supplementary Figure S7 — PTSL expression arrests LUAD cells in G2 regardless of p53 status [file crc-22-0473-s07.pdf]

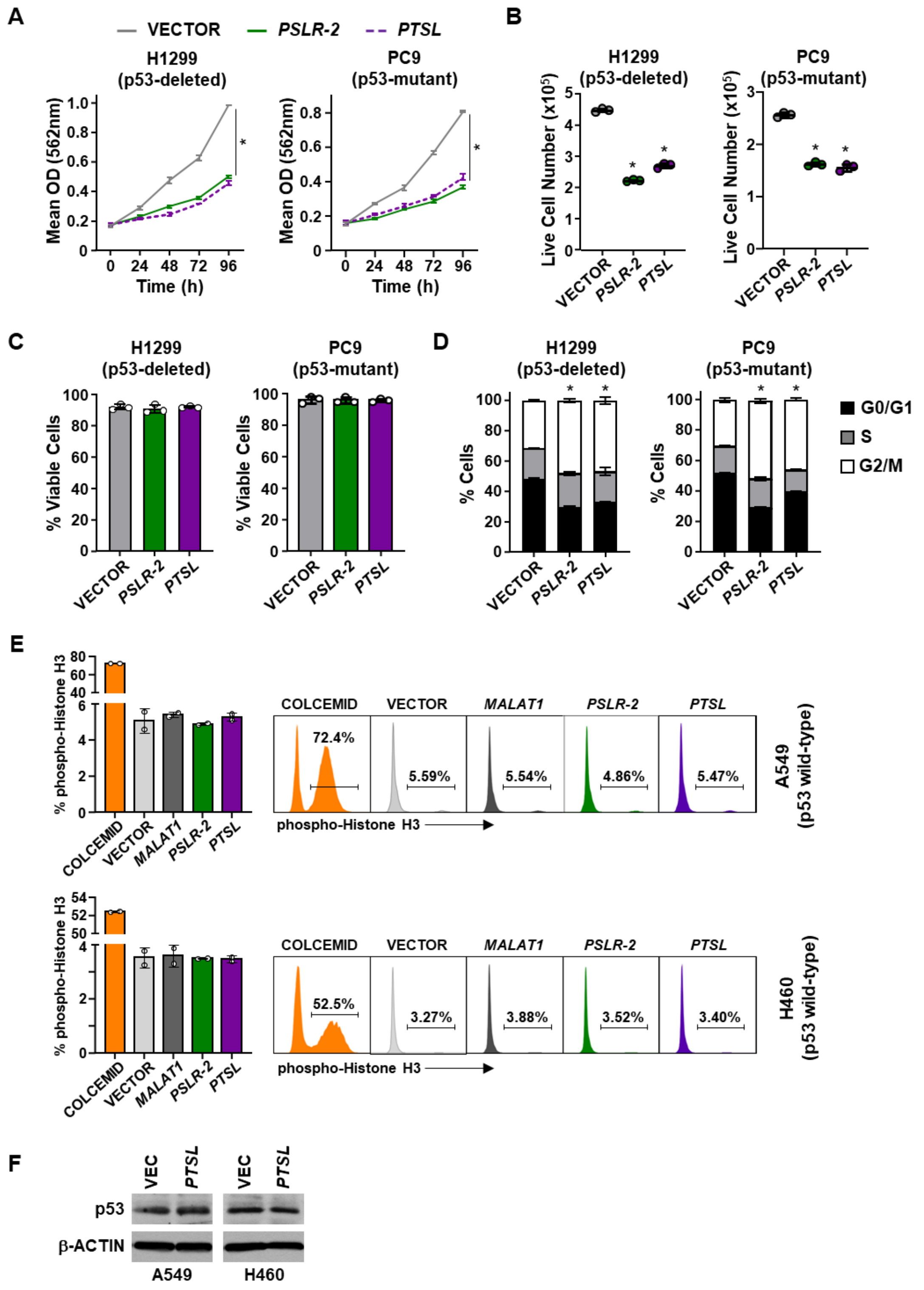

Supplementary Figure S7. *PTSL* expression arrests LUAD cells in G2 regardless of p53 status. The lncRNAs *PTSL* or *PSLR-2* or vector control were expressed (lentivirus) in LUAD cell lines. A) MTT assays (quadruplicate) performed at 24hr intervals in H1299

(*p53* deleted) and PC9 (p53 mutant) cells. Each assay was performed 2 independent times for both cell lines and one representative experiment is shown; mean ±SD; H1299 **P*<0.6x10^{-4} and PC9 **P*<0.1x10^{-4}. B and C) Trypan Blue dye exclusion was performed 24hr after lentiviral infection of H1299 and PC9 cells to determine live cell number (B) and viability (C). Each assay was performed 2 independent times for both cell lines and one representative experiment is shown; mean ±SD; for live cell number, **P*<0.1x10^{-4} for both H1299 and PC9. D) Following propidium iodide intercalation into DNA, cell cycle analysis was performed, in triplicate, by flow cytometry 24hr after lentiviral infection; mean ±SD; H1299 **P*<0.037 and PC9 **P*<0.015. E) p53 wild-type expressing LUAD cells, A549 and H460, expressing *PTSL*, *PSLR-2*, *MALAT1*, or empty vector control via lentiviral infection. The percentage of cells in mitosis was determined with intracellular detection of phospho-Histone H3, a mitotic marker, 2 independent times in both cell lines, in duplicate. Quantification (left; mean±SD) and representative histograms (right) from one experiment are shown. Colcemid-treated cells that arrest in mitosis were used as a positive control. F) Western blotting for p53 protein was performed 48hr after lentiviral expression of *PTSL* or vector control in A549 and H460 cells.
