# Supplementary material for: Decoding the lncRNAome Across Diverse Cellular Stresses Reveals Core p53-effector Pan-cancer Suppressive lncRNAs
Source: Cancer Res Commun. 2023 May 11;3(5):842–59. doi: 10.1158/2767-9764.CRC-22-0473 (PMC10173889; doi:10.1158/2767-9764.CRC-22-0473)
Supplement: Supplementary Table S1 — Summary of 14 RNA-seq and 23 ChIP-seq datasets evaluated [file crc-22-0473-s08.pdf]

Supplemental Tables

Supplementary Table S1. Summary of 14 RNA-seq and 23 ChIP-seq datasets evaluated.

Data type	GEO ID	Treatment	Cells/Cell line	PubMed ID
RNA sequencing data	GSE111009	DMSO vs Nutlin	Foreskin fibroblast	31113863
	GSE111009	DMSO vs Nutlin	MCF10A	31113863
	GSE124508	DMSO vs Idasanutlin/RG7388[a]	TTC642	30755442
	GSE128191	DMSO vs Nutlin	Neural crest cells	31178404
	GSE47042	Unstimulated vs Nutlin	MCF7	25058159
	GSE80716	DMSO vs Nutlin	MCF7	27183917
	GSE87668	DMSO vs Nutlin	U2OS	27829155
	GSE110387	Untreated vs Ionizing radiation	U2OS	29476964; 30419821
	GSE55727	Untreated vs Doxorubicin	GM06170	25883152
	GSE55727	Untreated vs Doxorubicin	GM00011	25883152
	GSE79249	Untreated vs Doxorubicin	HCT116	28877474
	GSE78512	Untreated vs 5-Fluorouracil at 24 hr	MCF7	27602759
	GSE78512	Untreated vs 5-Fluorouracil at 48 hr	MCF7	27602759
	GSE89807	DMSO vs 5-Fluorouracil	SJSA	28416637
**Data type**	**ChIPBase ID**	**Treatment**	**Cells/Cell line**	**PubMed ID**
ChIP sequencing data	HUMHG03463	DMSO (Vehicle)	IMR90	25391375
	HUMHG02401	DMSO (Vehicle)	U2OS	23775793
	HUMHG02404	Untreated	U2OS	23775793
	HUMHG03325	Untreated	Keratinocytes	24823795
	HUMHG03331	Untreated	Keratinocytes	24823795
	HUMHG02422	Untreated	GM06993, GM11992	24120139
	HUMHG02423	Nutlin	GM12878	24120139
	HUMHG03464	Nutlin	IMR90	25391375
	HUMHG02403	Nutlin	U2OS	23775793
	HUMHG03452	5-Fluorouracil	HCT116	25415302
	HUMHG01701	5-Fluorouracil	IMR90	22127205
	HUMHG01497	Actinomycin D	U2OS	21394211
	HUMHG01499	Actinomycin D	U2OS	21394211
	HUMHG03327	Cisplatin	Keratinocytes	24823795
	HUMHG03333	Cisplatin	Keratinocytes	24823795
	HUMHG01231	Doxorubicin	GM06170, GM00011	25883152
	HUMHG02421	Doxorubicin	GM06993, GM11992	24120139
	HUMHG03326	Doxorubicin	Keratinocytes	24823795
	HUMHG03332	Doxorubicin	Keratinocytes	24823795
	HUMHG02402	Doxorubicin	U2OS	23775793
	HUMHG01498	Etoposide	U2OS	21394211
	HUMHG01500	Etoposide	U2OS	21394211
	HUMHG01502	Etoposide	U2OS	21394211

[a]Nutlin derivative
