# Supplementary material for: Decoding the lncRNAome Across Diverse Cellular Stresses Reveals Core p53-effector Pan-cancer Suppressive lncRNAs
Source: Cancer Res Commun. 2023 May 11;3(5):842–59. doi: 10.1158/2767-9764.CRC-22-0473 (PMC10173889; doi:10.1158/2767-9764.CRC-22-0473)
Supplement: Supplementary Table S2 — TCGA cancer types evaluated to identify differentially expressed lncRNAs in samples with p53LOF compared with p53WT [file crc-22-0473-s09.pdf]

Supplementary Table S2. TCGA cancer types evaluated to identify differentially expressed lncRNAs in samples with $p53^{LOF}$ compared with $p53^{WT}$.

Cancer type	TCGA ID	Number of samples					
		mRNA	Methyl-ation	#CNA	lncRNA	*$p53^{WT}$	**$p53^{LOF}$
Bladder urothelial carcinoma	BLCA	407	413	408	252	126	36
Breast invasive carcinoma	BRCA	1104	790	1080	837	538	111
Head and neck squamous cell carcinoma	HNSC	522	530	522	426	132	119
Kidney renal clear cell carcinoma	KIRC	534	320	528	448	421	3
Liver hepatocellular carcinoma	LIHC	373	379	370	200	139	19
Lung adenocarcinoma	LUAD	517	460	516	488	235	92
Ovarian carcinoma	OV	308	602	579	412	147	102
Skin cutaneous melanoma	SKCM	473	474	367	226	187	13
Stomach adenocarcinoma	STAD	380	396	441	285	154	47
Uterine corpus endometrial carcinoma	UCEC	370	432	539	316	215	24

#copy number alteration; *WT: wild-type; **LOF: loss of function
