# Supplementary material for: Decoding the lncRNAome Across Diverse Cellular Stresses Reveals Core p53-effector Pan-cancer Suppressive lncRNAs
Source: Cancer Res Commun. 2023 May 11;3(5):842–59. doi: 10.1158/2767-9764.CRC-22-0473 (PMC10173889; doi:10.1158/2767-9764.CRC-22-0473)
Supplement: Supplementary Table S3 — Number of cell lines of the indicated cancer types with CRISPR or RNAi screening data [file crc-22-0473-s10.pdf]

Supplementary Table S3. Number of cell lines of the indicated cancer types with CRISPR or RNAi screening data.

Cancer type	RNAi	CRISPR
BLCA	12	27
BRCA	80	29
HNSC	19	27
KIRC	7	6
LIHC	16	19
LUAD	55	34
OV	37	36
SKCM	46	37
STAD	24	22
UCEC	15	18
